# Supplementary figures and images for: Interrogating the structure and function of the human voltage-gated proton channel (hHv1) with a fluorescent noncanonical amino acid
Source: eLife. 2026 Apr 28;15:RP110161. doi: 10.7554/eLife.110161 (PMC13124150; doi:10.7554/eLife.110161)

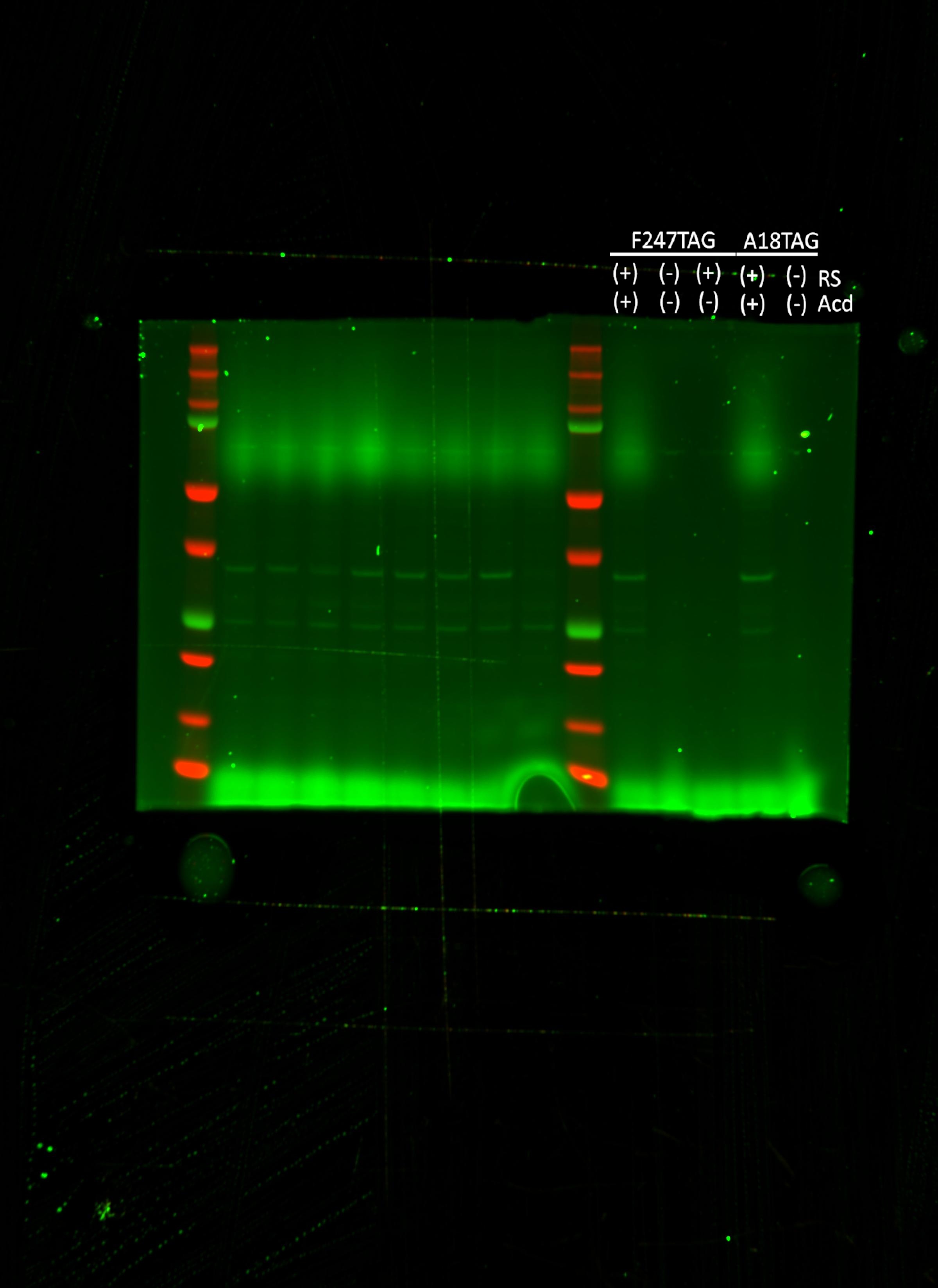

Supplement: Figure 1—source data 1. [file elife-110161-fig1-data1.zip › hHv1-TAG expression Acd fluorescence Labeled.jpg]

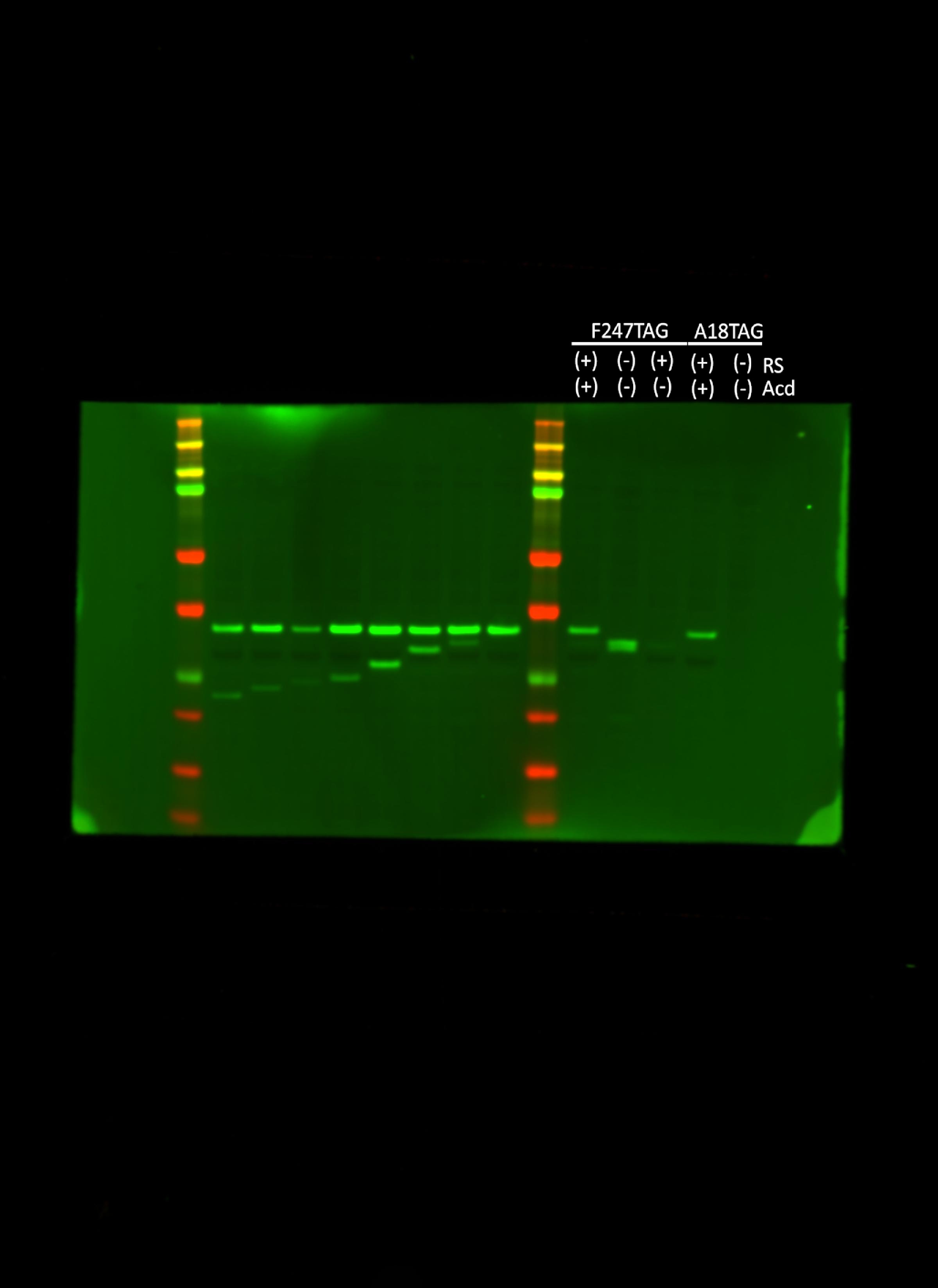

Supplement: Figure 1—source data 1. [file elife-110161-fig1-data1.zip › hHv1-TAG expression WB Labeled.jpg]

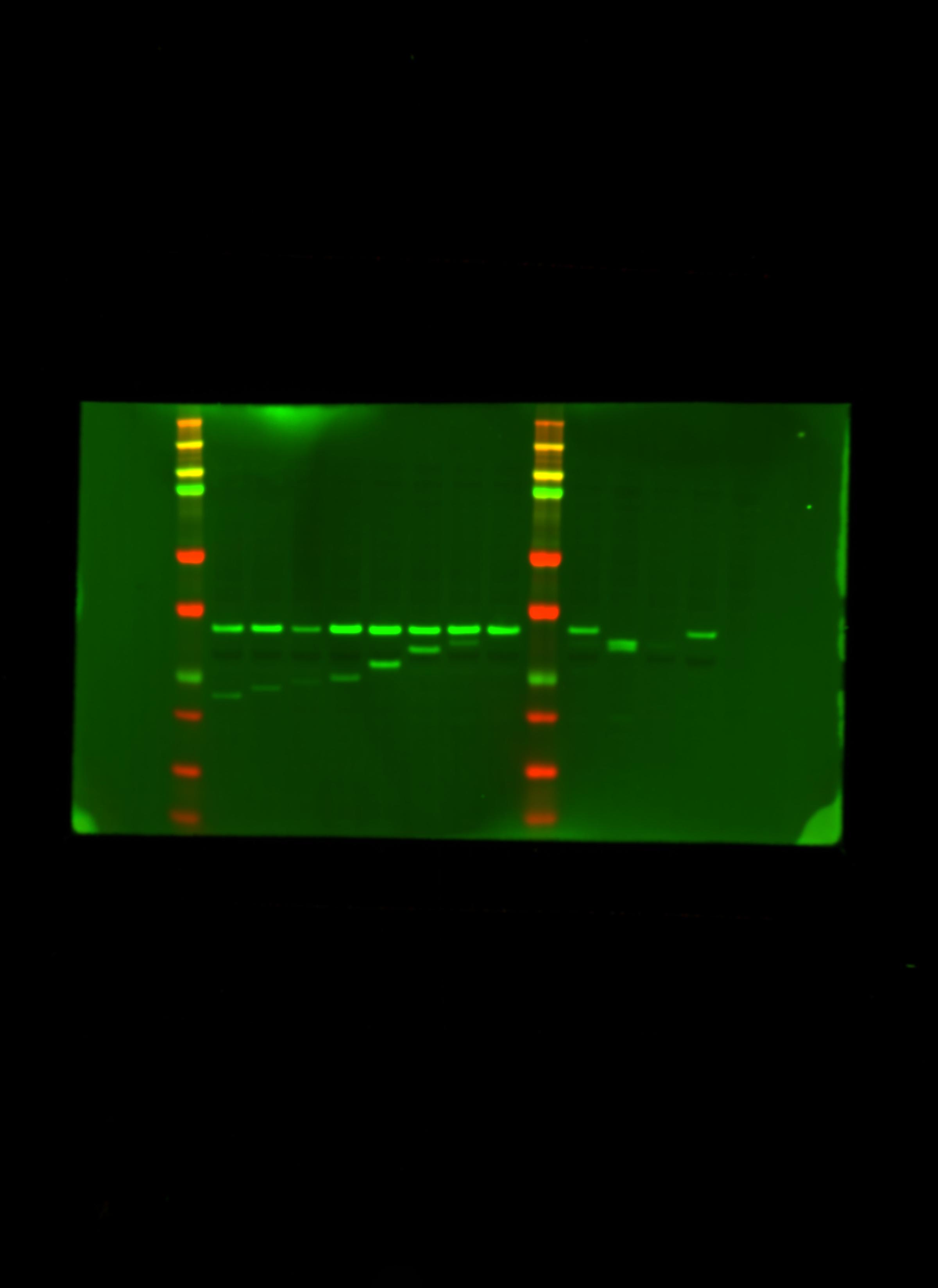

Supplement: Figure 1—source data 2. [file elife-110161-fig1-data2.zip › hHv1-TAG expression WB.jpg]

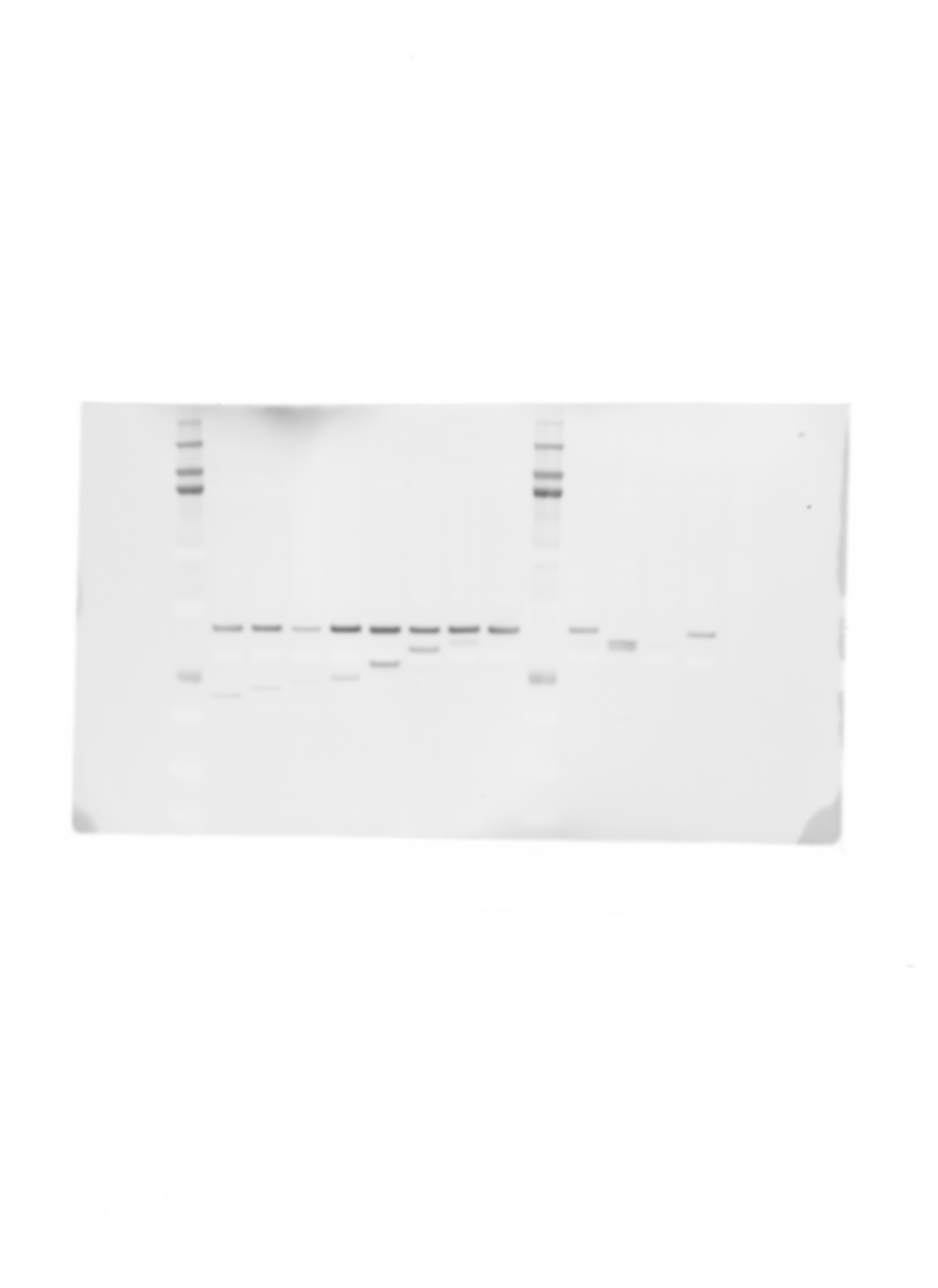

Supplement: Figure 1—source data 2. [file elife-110161-fig1-data2.zip › hHv1-TAG expression WB.tif]

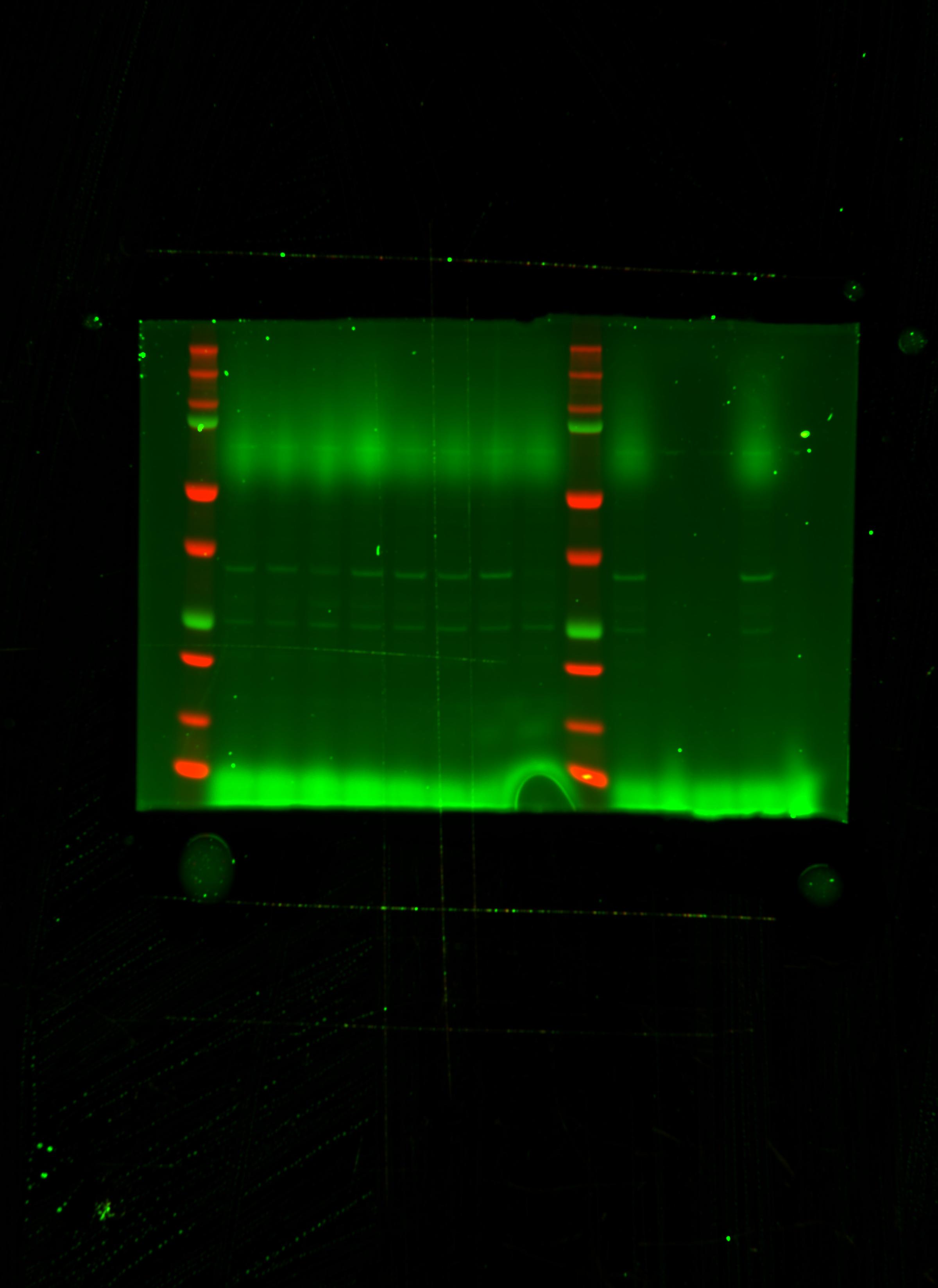

Supplement: Figure 1—source data 2. [file elife-110161-fig1-data2.zip › hHv1-TAG expression Acd fluorescence.jpg]

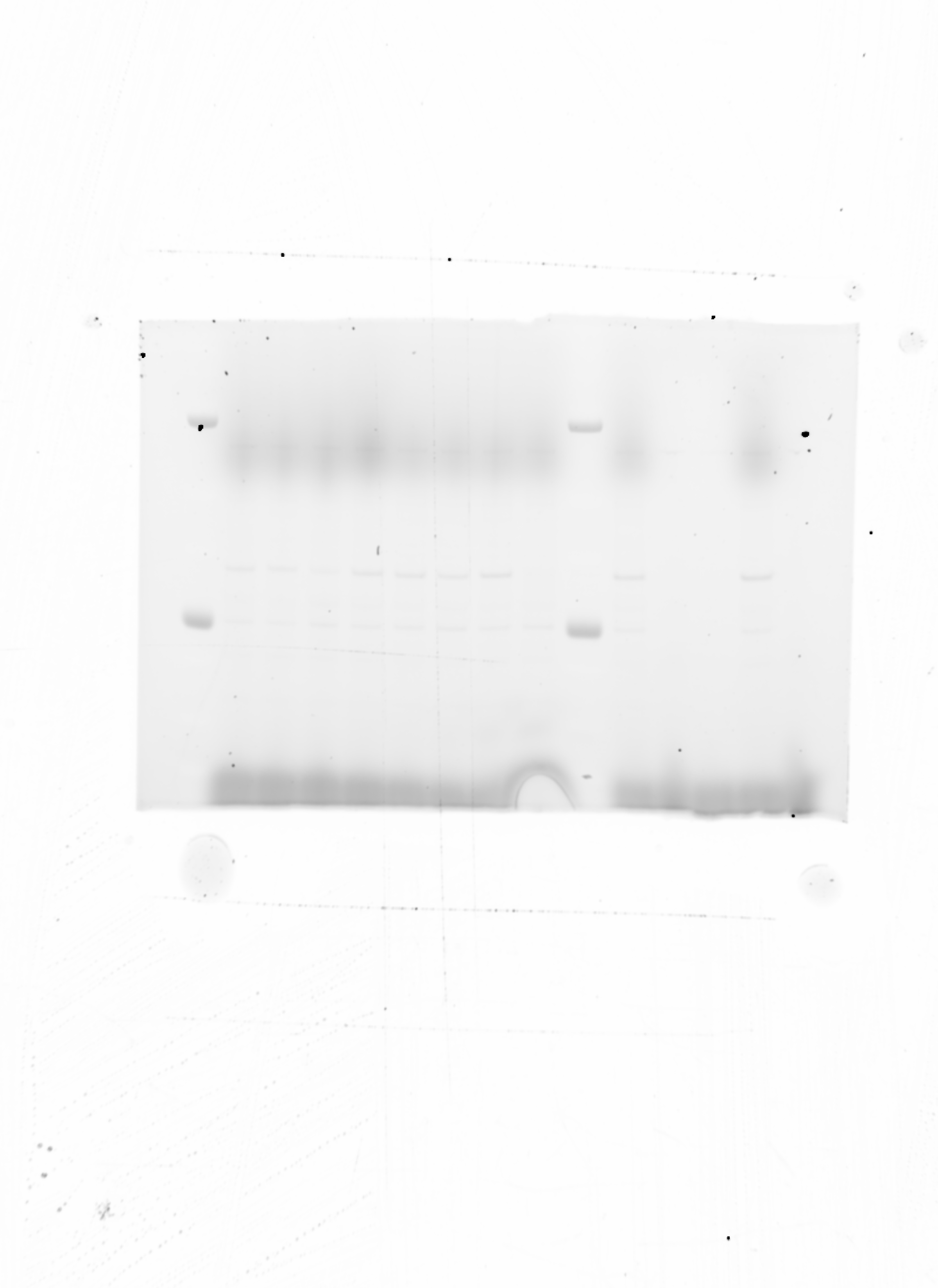

Supplement: Figure 1—source data 2. [file elife-110161-fig1-data2.zip › hHv1-TAG expression Acd fluorescence.tif]

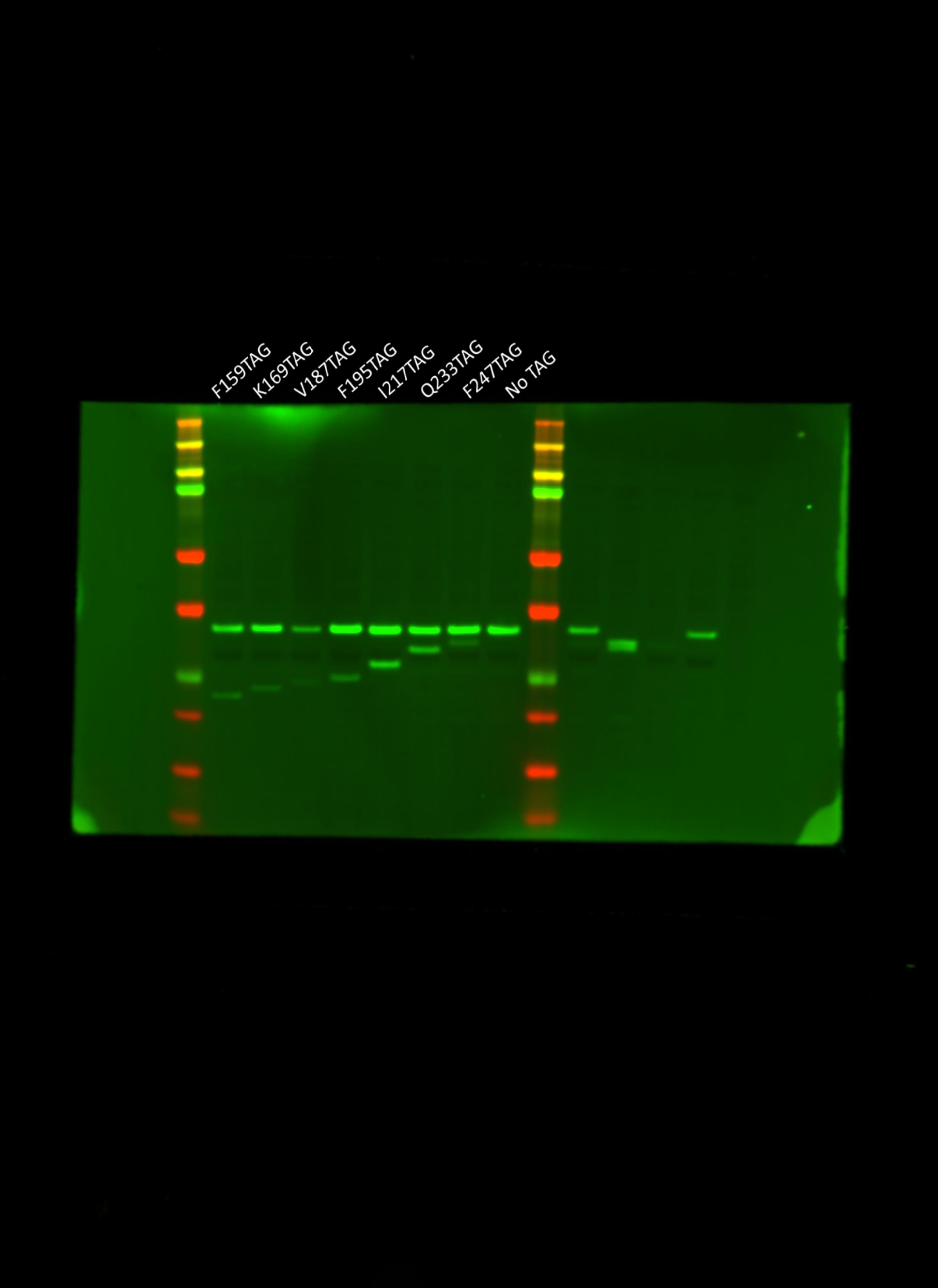

Supplement: Figure 2—source data 1. [file elife-110161-fig2-data1.zip › hHv1-TAG2mutants WB Labeled.jpg]

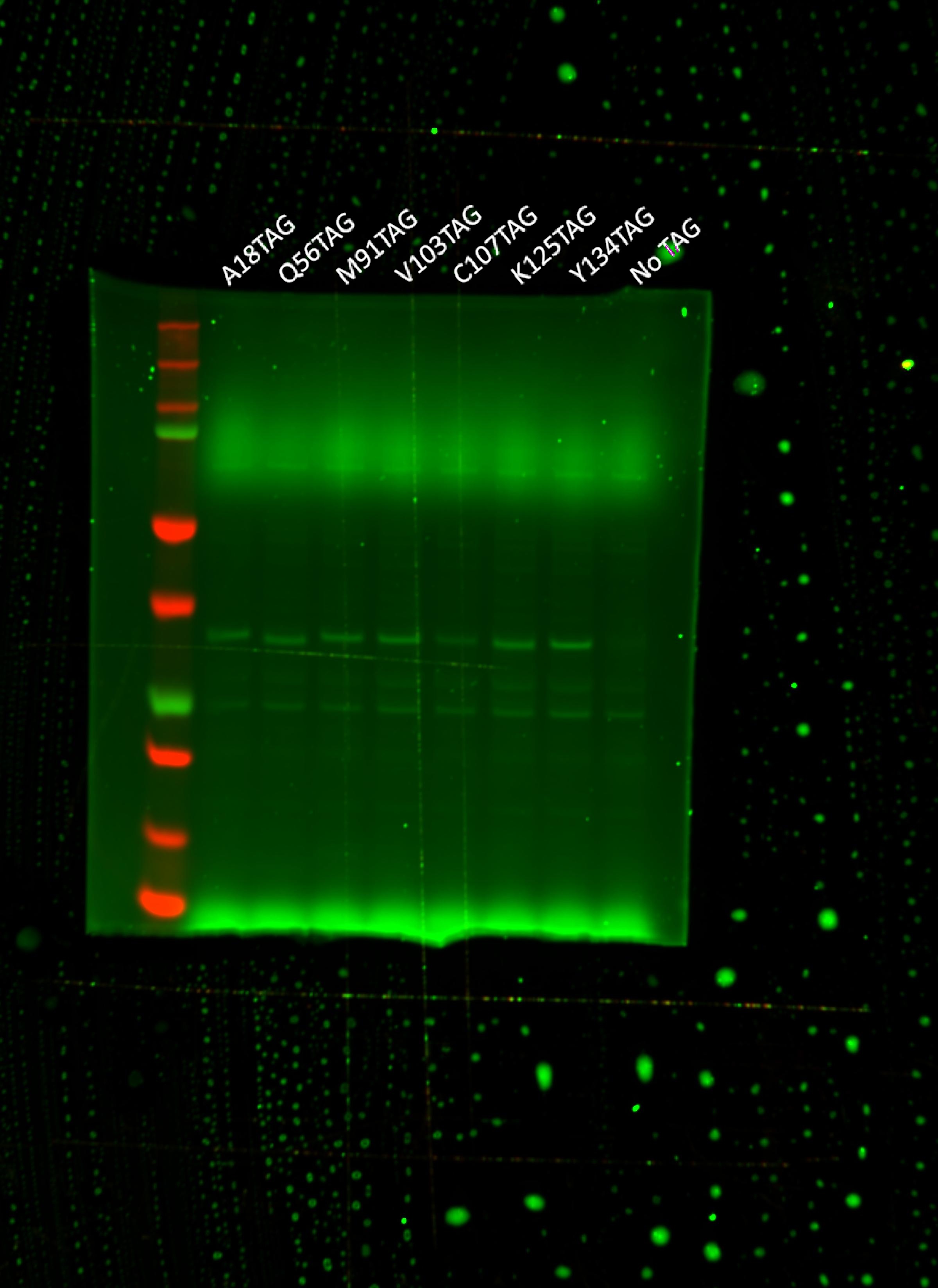

Supplement: Figure 2—source data 1. [file elife-110161-fig2-data1.zip › hHv1-TAG1mutants Acd fluorescence Labeled.jpg]

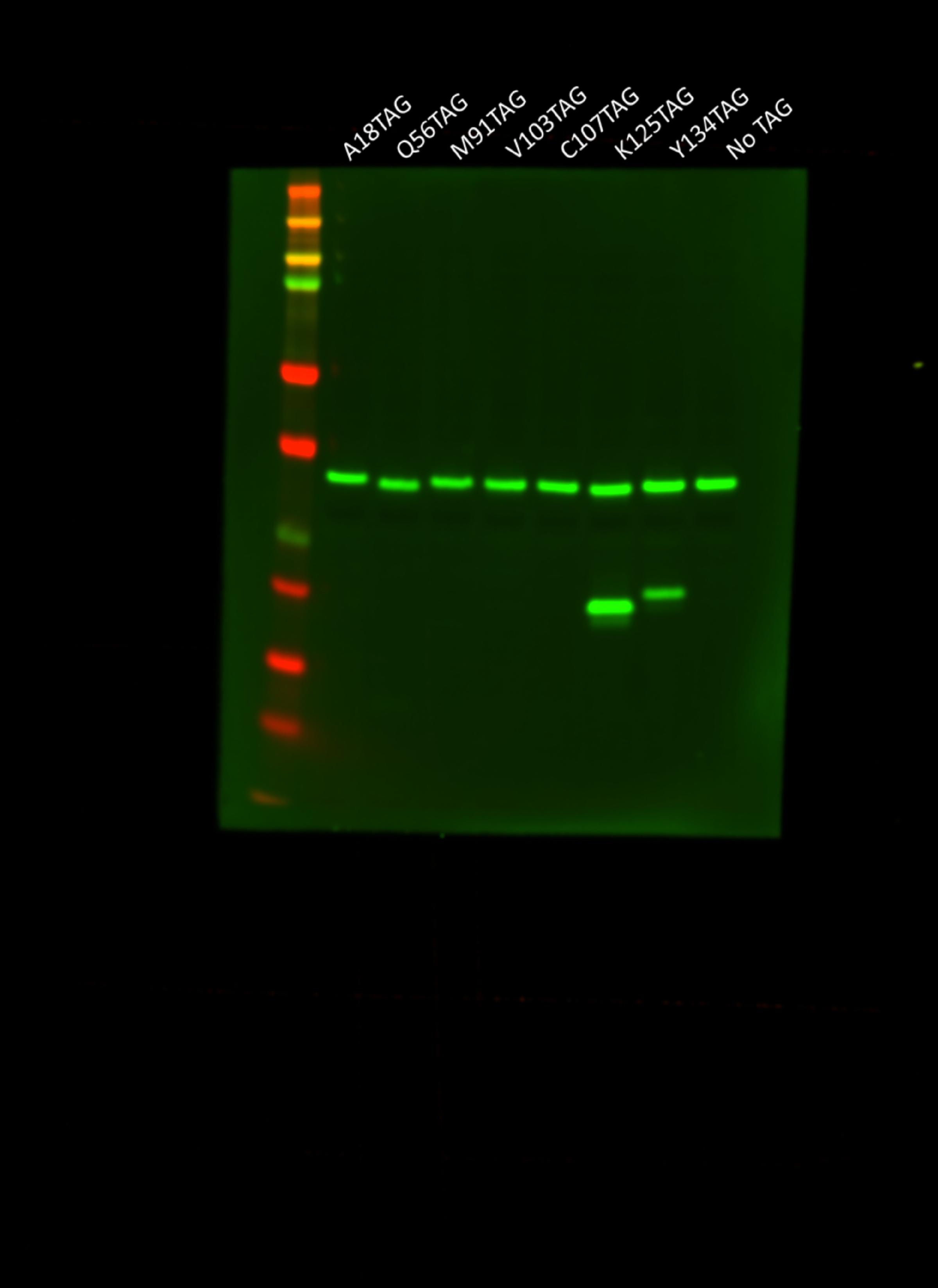

Supplement: Figure 2—source data 1. [file elife-110161-fig2-data1.zip › hHv1-TAG1mutants WB Labeled.jpg]

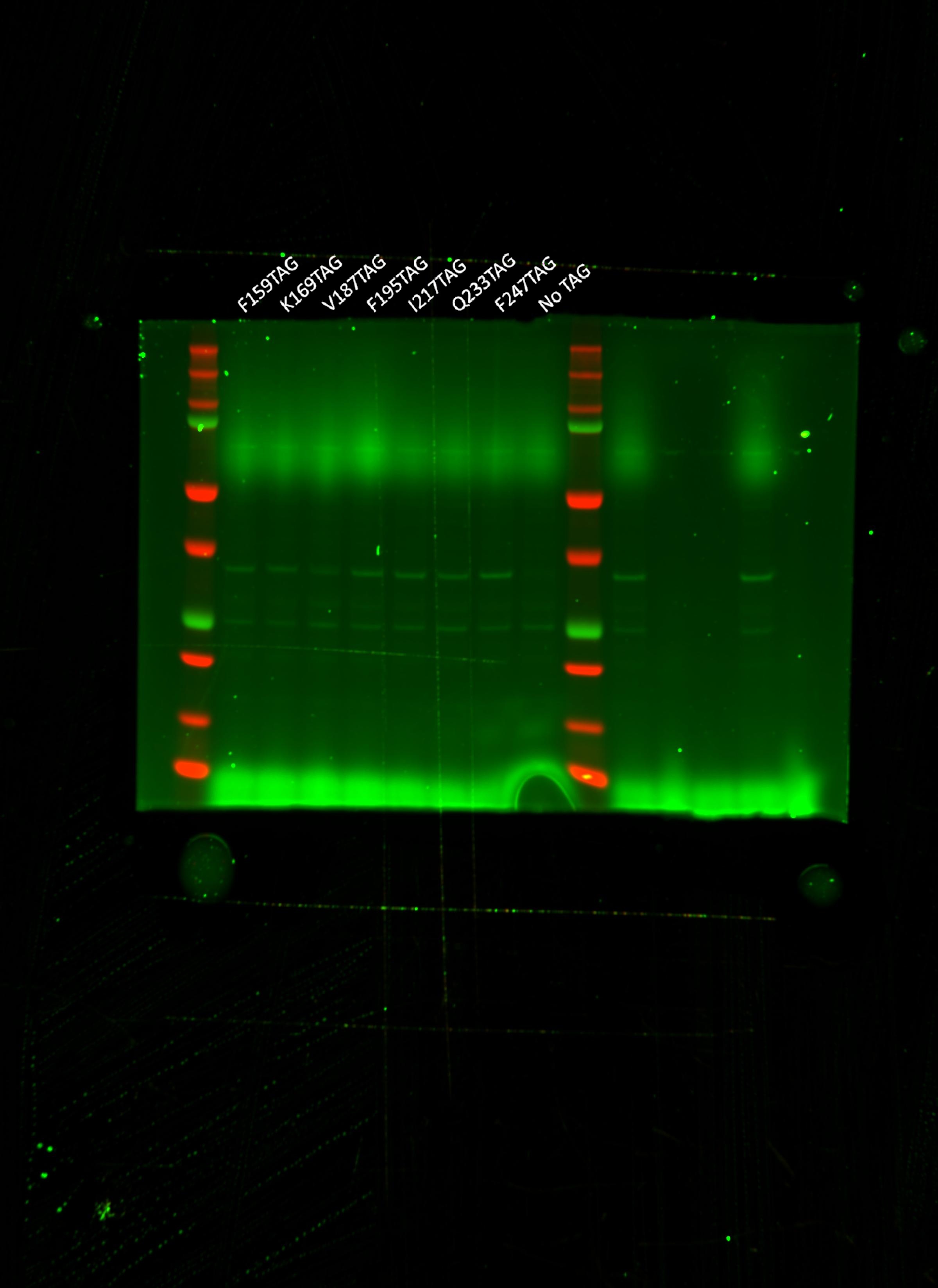

Supplement: Figure 2—source data 1. [file elife-110161-fig2-data1.zip › hHv1-TAG2mutants Acd fluorescence Labeled.jpg]

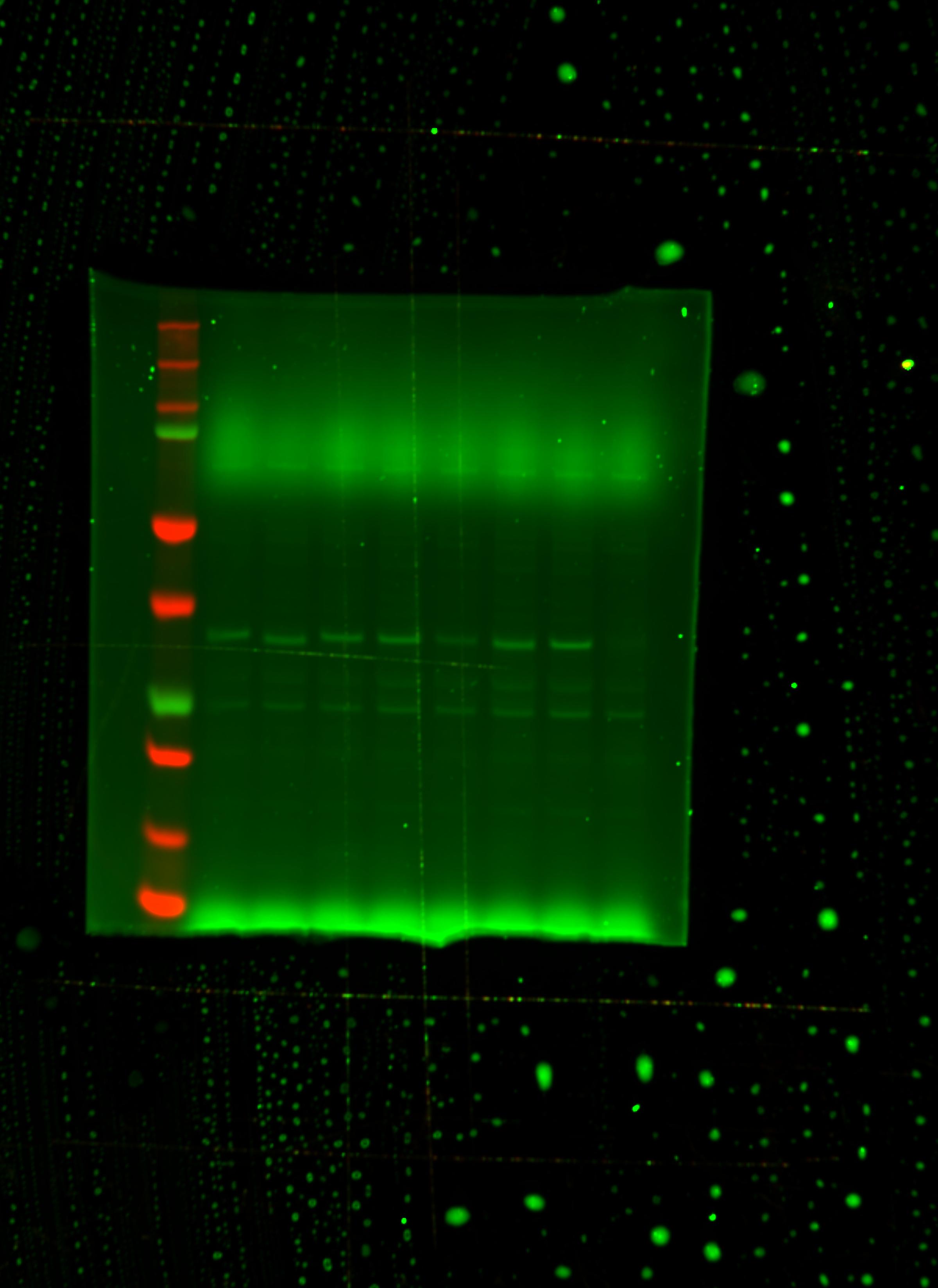

Supplement: Figure 2—source data 2. [file elife-110161-fig2-data2.zip › hHv1-TAG1mutants Acd fluorescence.jpg]

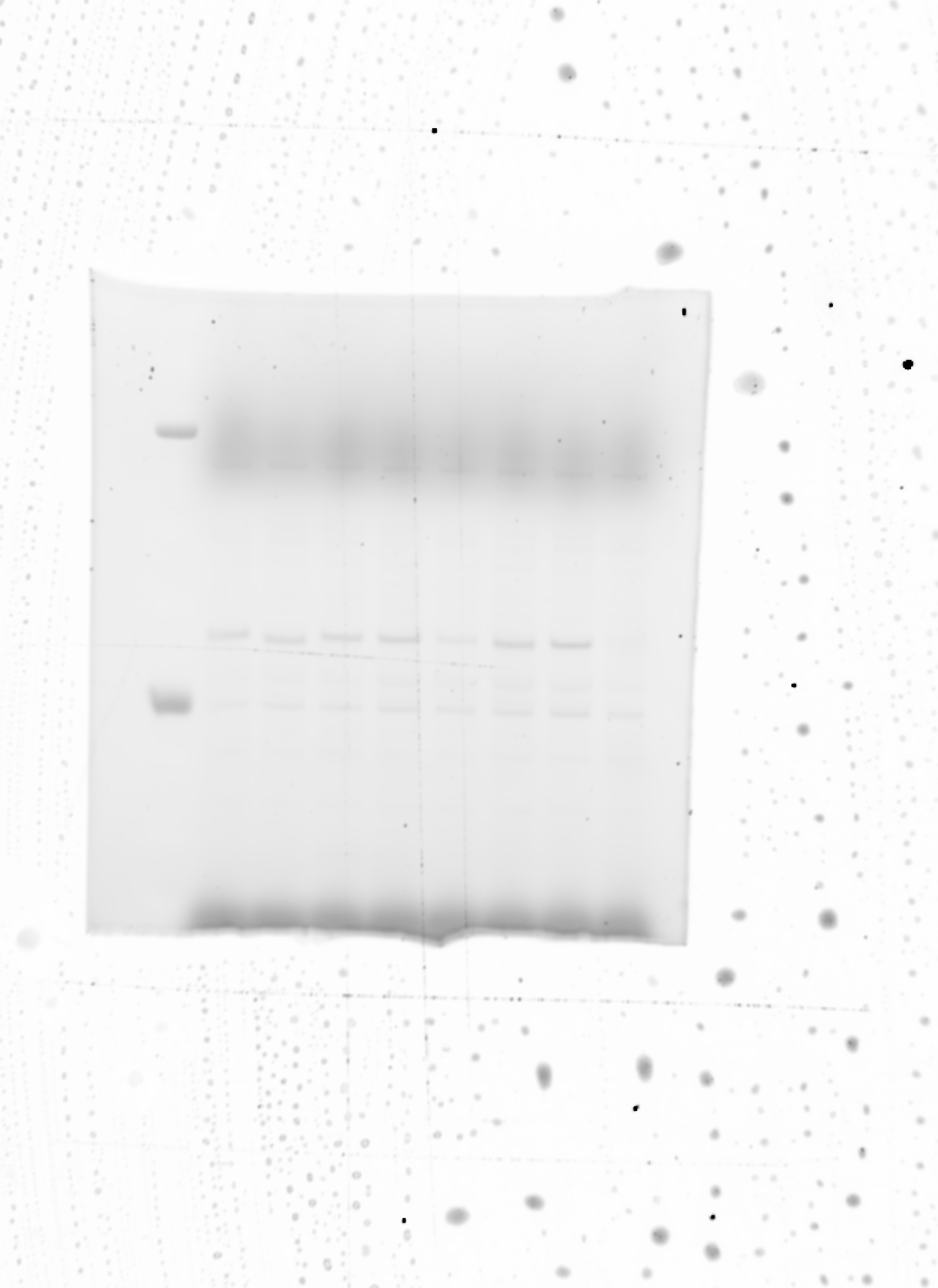

Supplement: Figure 2—source data 2. [file elife-110161-fig2-data2.zip › hHv1-TAG1mutants Acd fluorescence.tif]

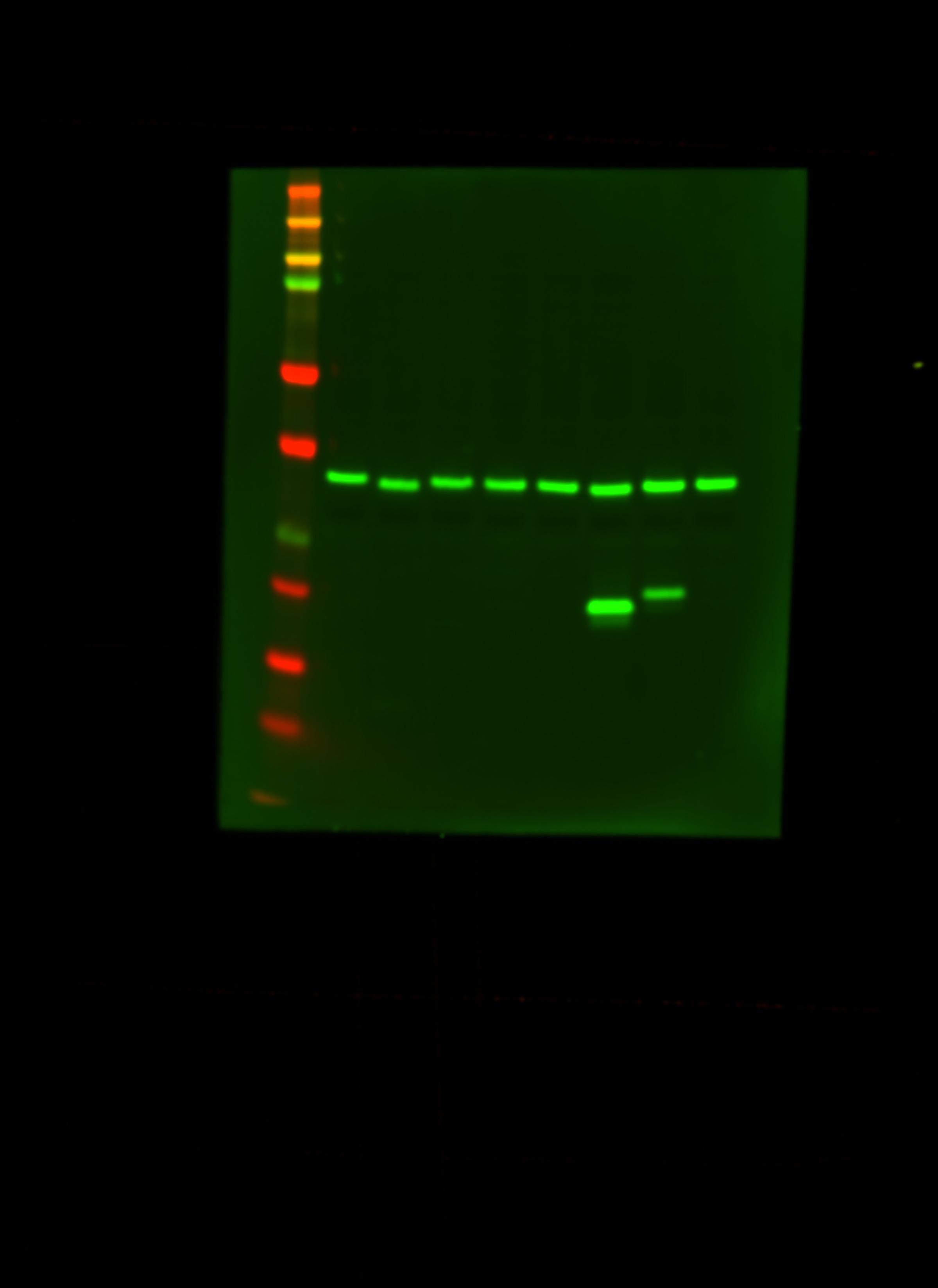

Supplement: Figure 2—source data 2. [file elife-110161-fig2-data2.zip › hHv1-TAG1mutants WB.jpg]

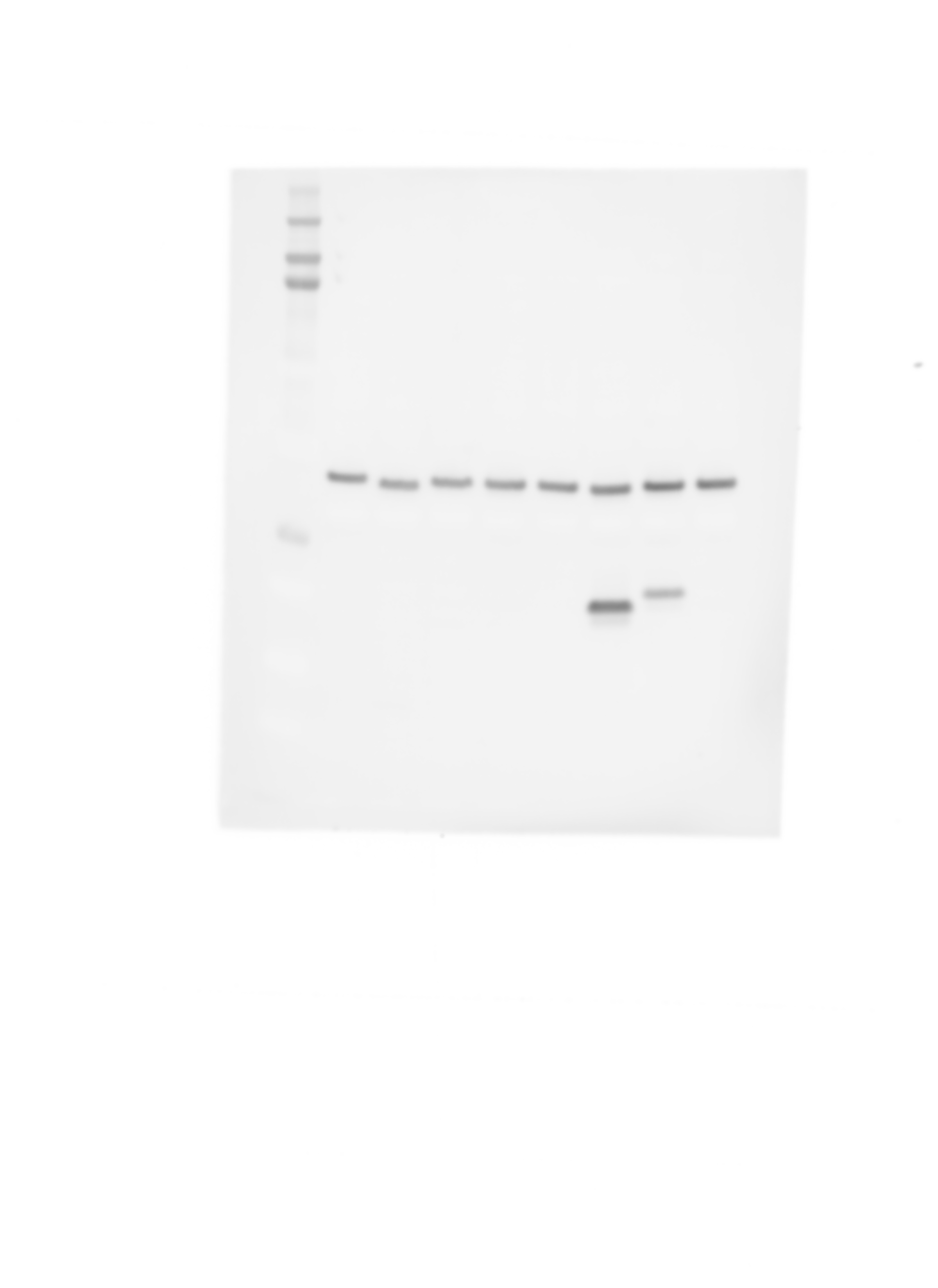

Supplement: Figure 2—source data 2. [file elife-110161-fig2-data2.zip › hHv1-TAG1mutants WB.tif]

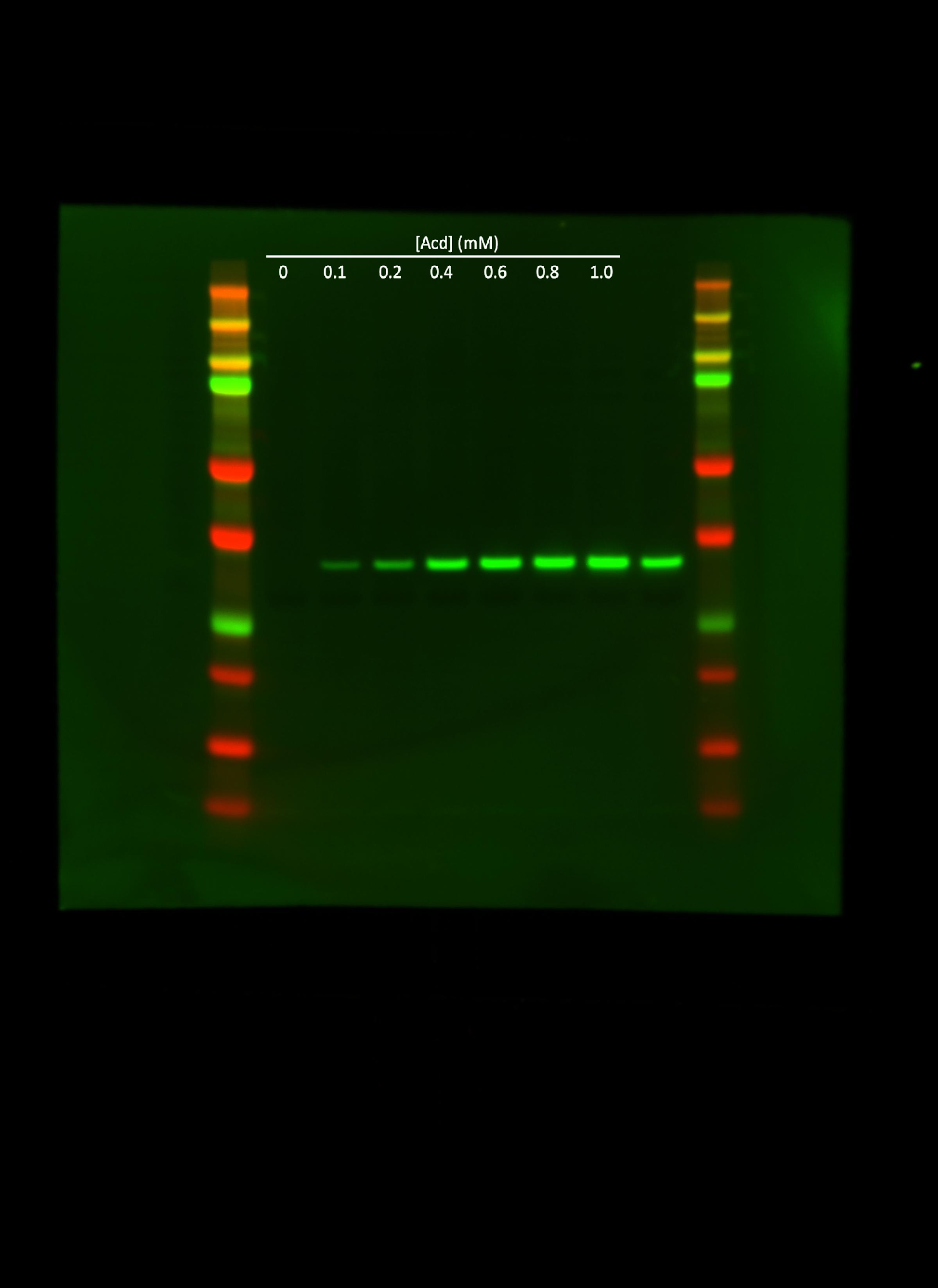

Supplement: Figure 2—figure supplement 2—source data 1. [file elife-110161-fig2-figsupp2-data1.zip › hHv1-A18TAG Acd WB Labeled.jpg]

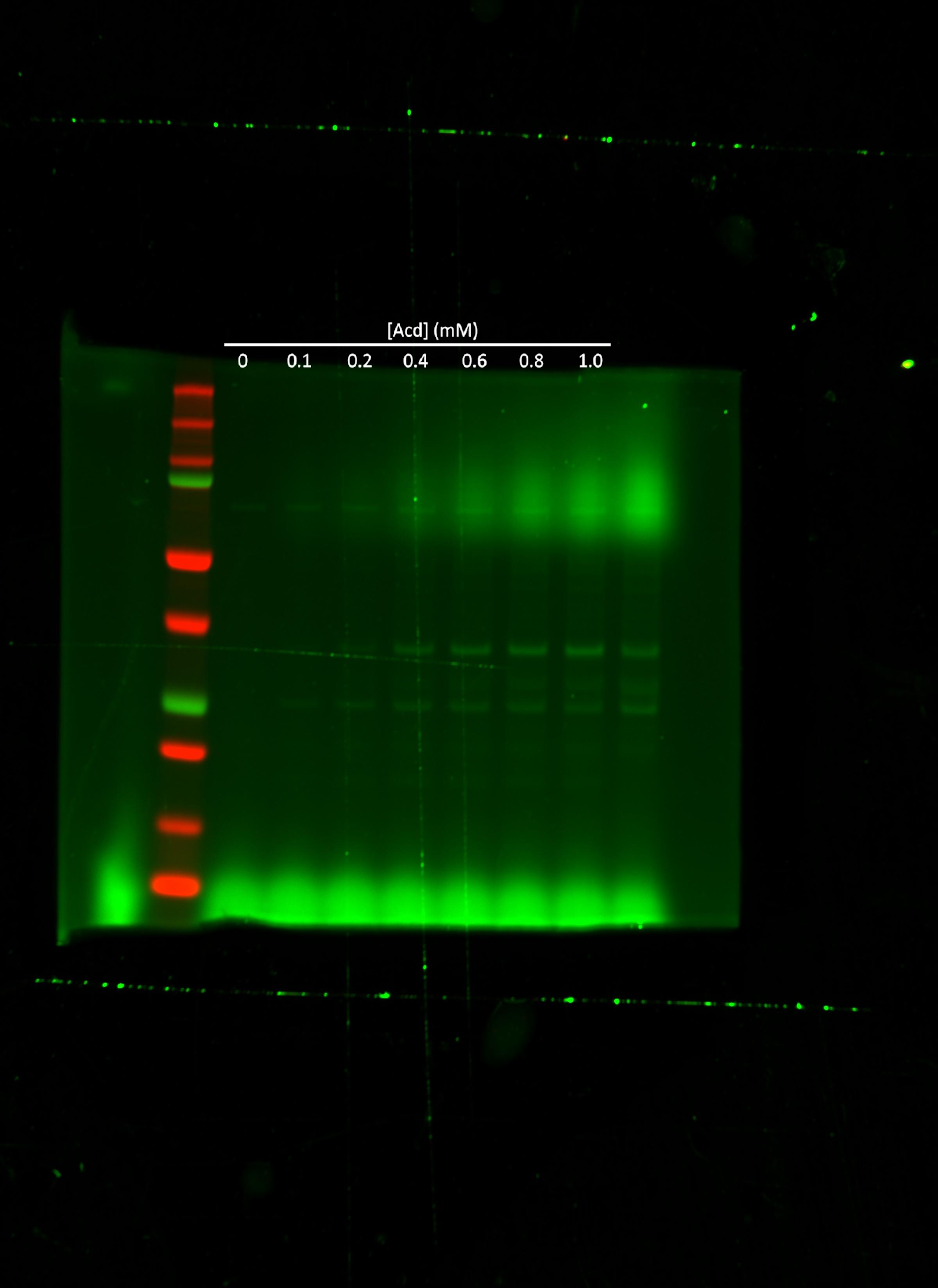

Supplement: Figure 2—figure supplement 2—source data 1. [file elife-110161-fig2-figsupp2-data1.zip › hHv1-A18TAG Acd fluorescence Labeled.jpg]

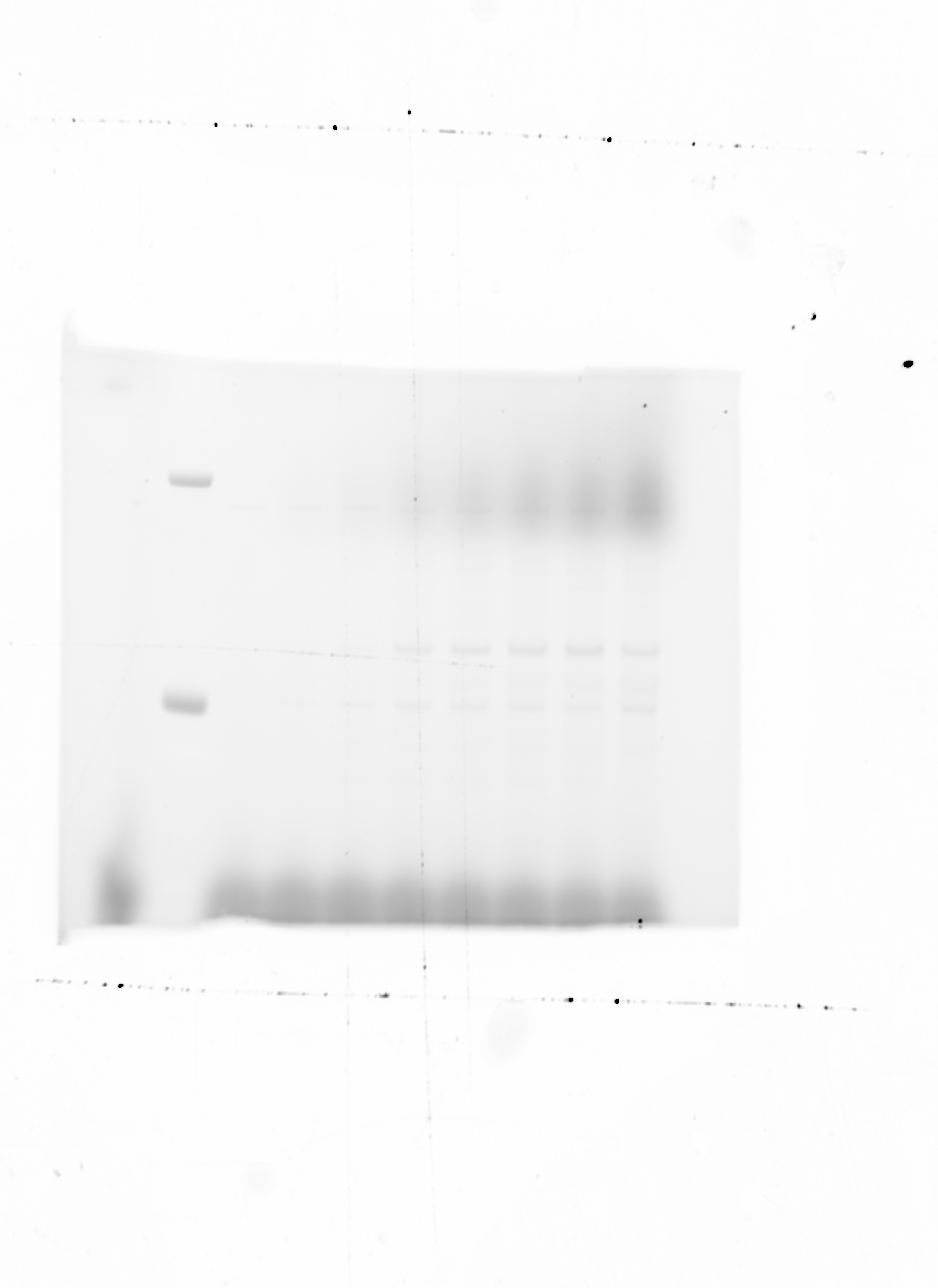

Supplement: Figure 2—figure supplement 2—source data 2. [file elife-110161-fig2-figsupp2-data2.zip › hHv1-A18TAG Acd fluorescence.tif]

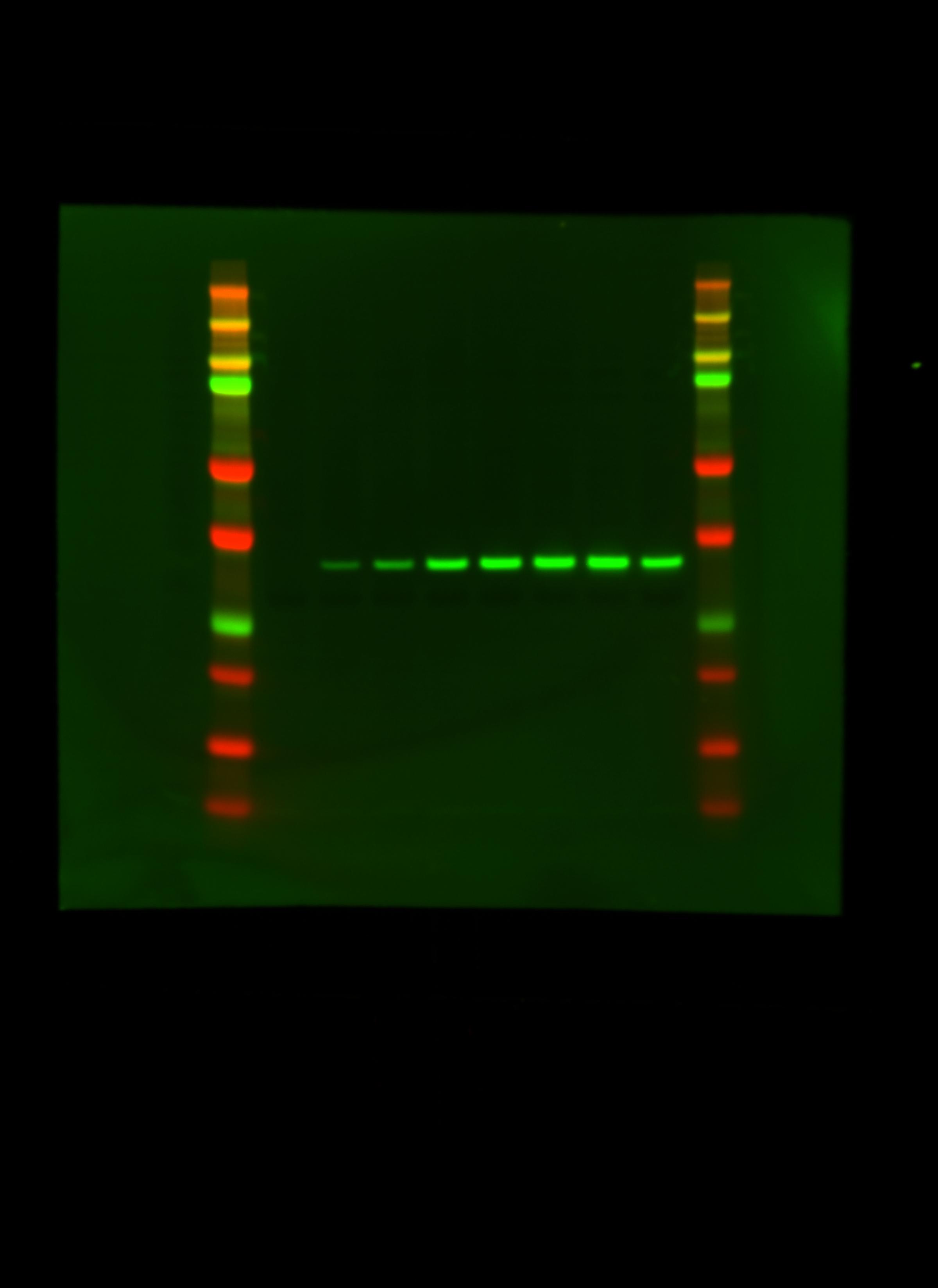

Supplement: Figure 2—figure supplement 2—source data 2. [file elife-110161-fig2-figsupp2-data2.zip › hHv1-A18TAG Acd WB.jpg]

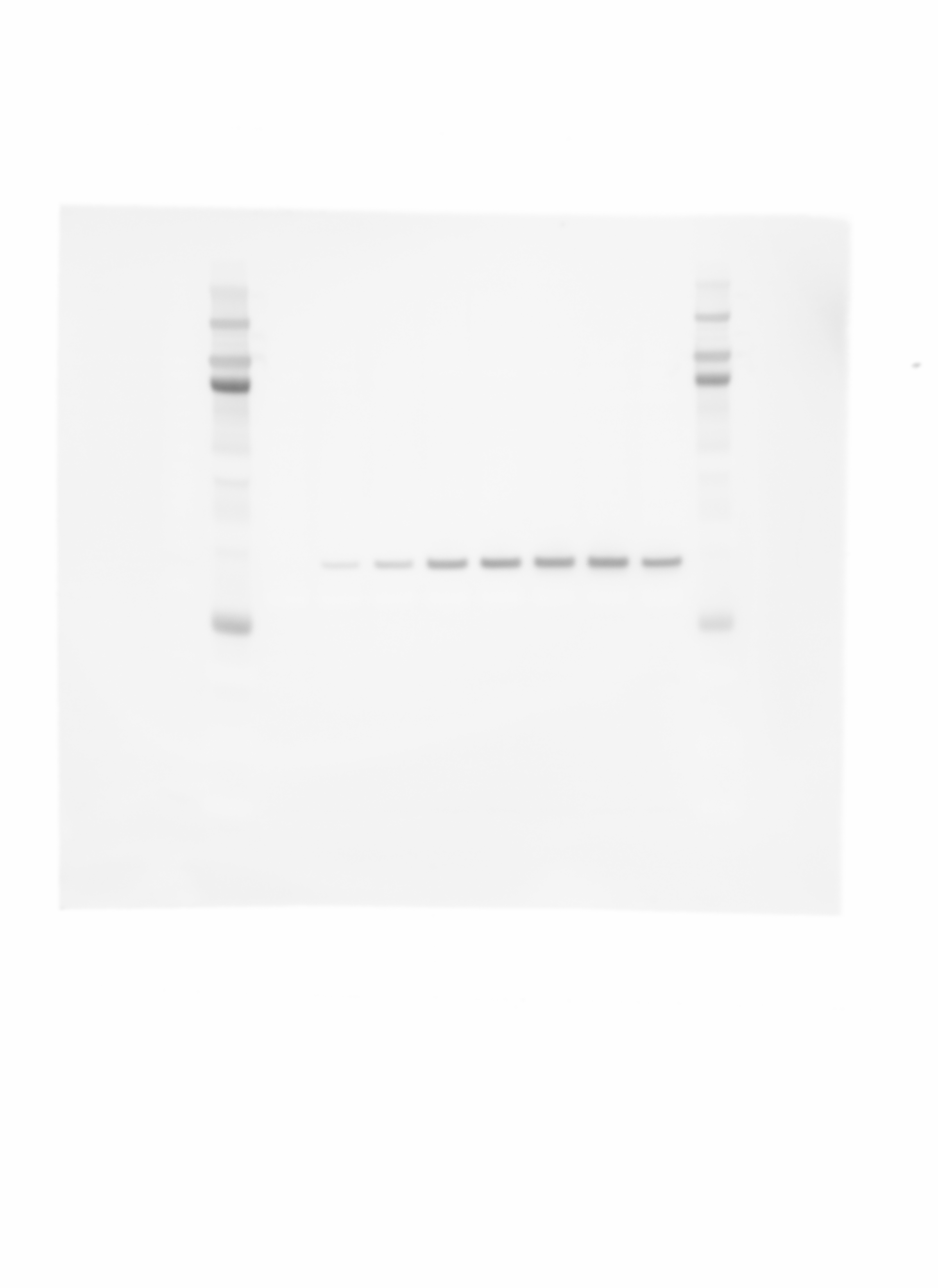

Supplement: Figure 2—figure supplement 2—source data 2. [file elife-110161-fig2-figsupp2-data2.zip › hHv1-A18TAG Acd WB.tif]

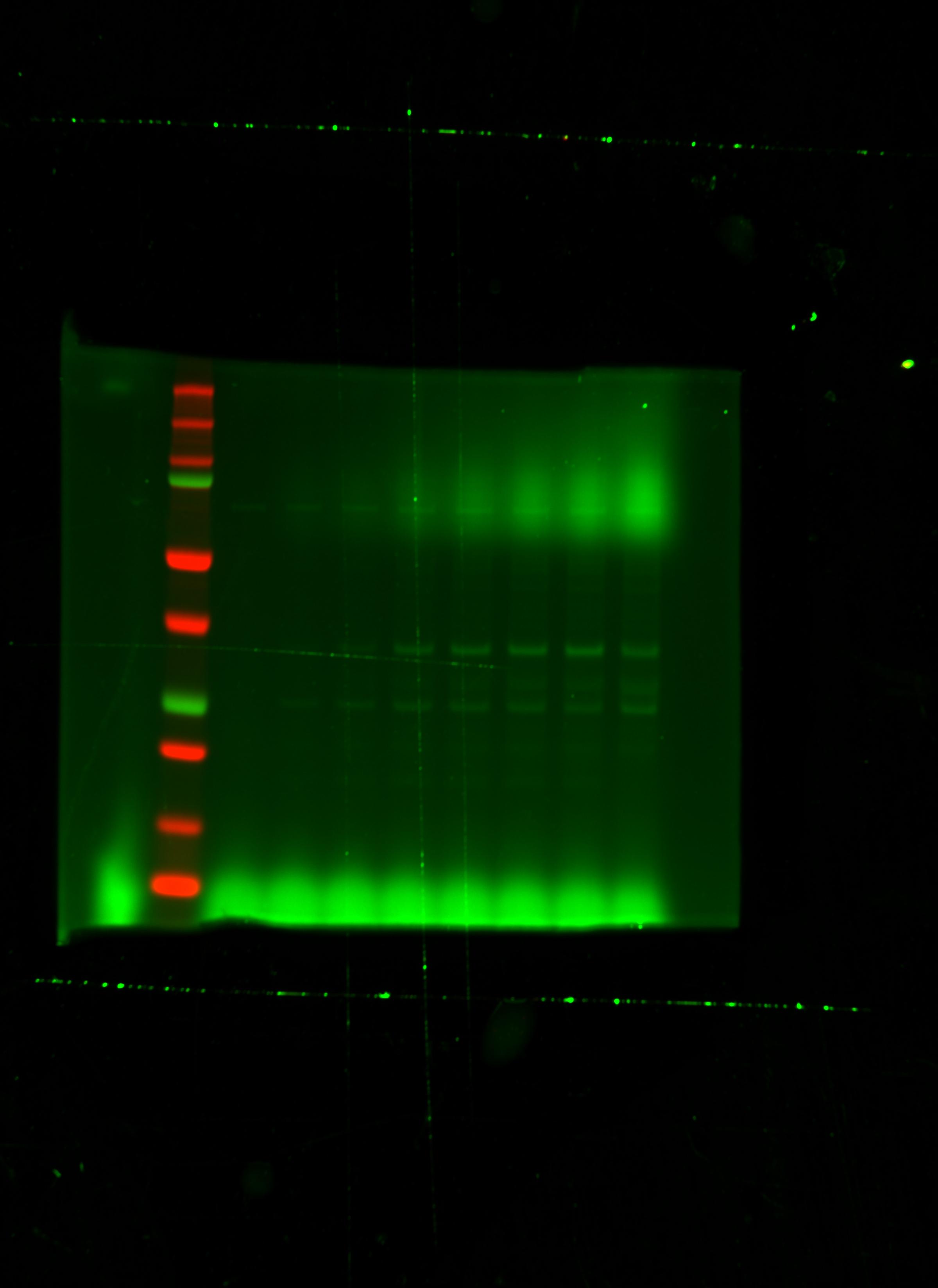

Supplement: Figure 2—figure supplement 2—source data 2. [file elife-110161-fig2-figsupp2-data2.zip › hHv1-A18TAG Acd fluorescence.jpg]

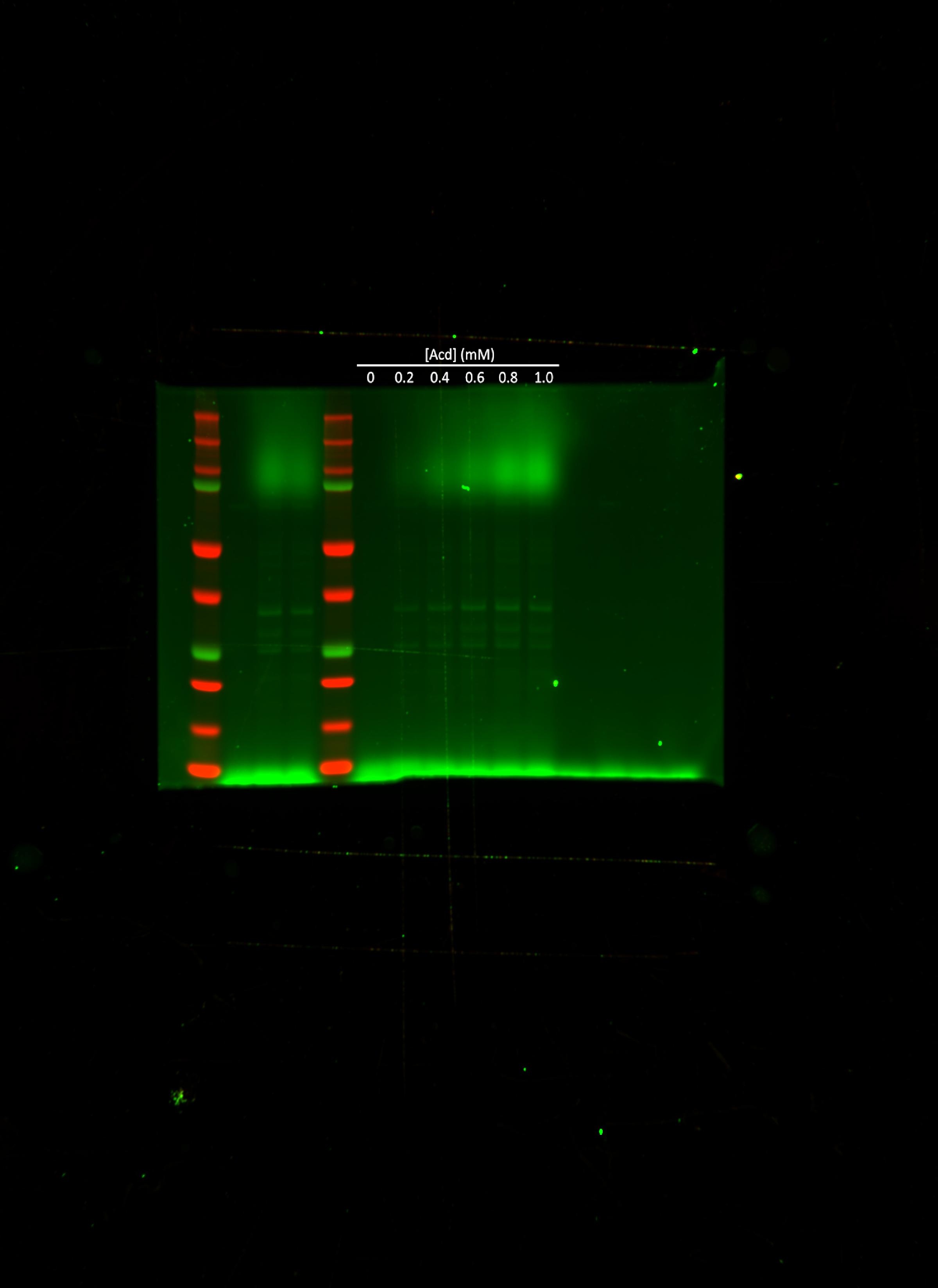

Supplement: Figure 2—figure supplement 3—source data 1. [file elife-110161-fig2-figsupp3-data1.zip › hHv1-K125TAG Acd fluorescence Labeled.jpg]

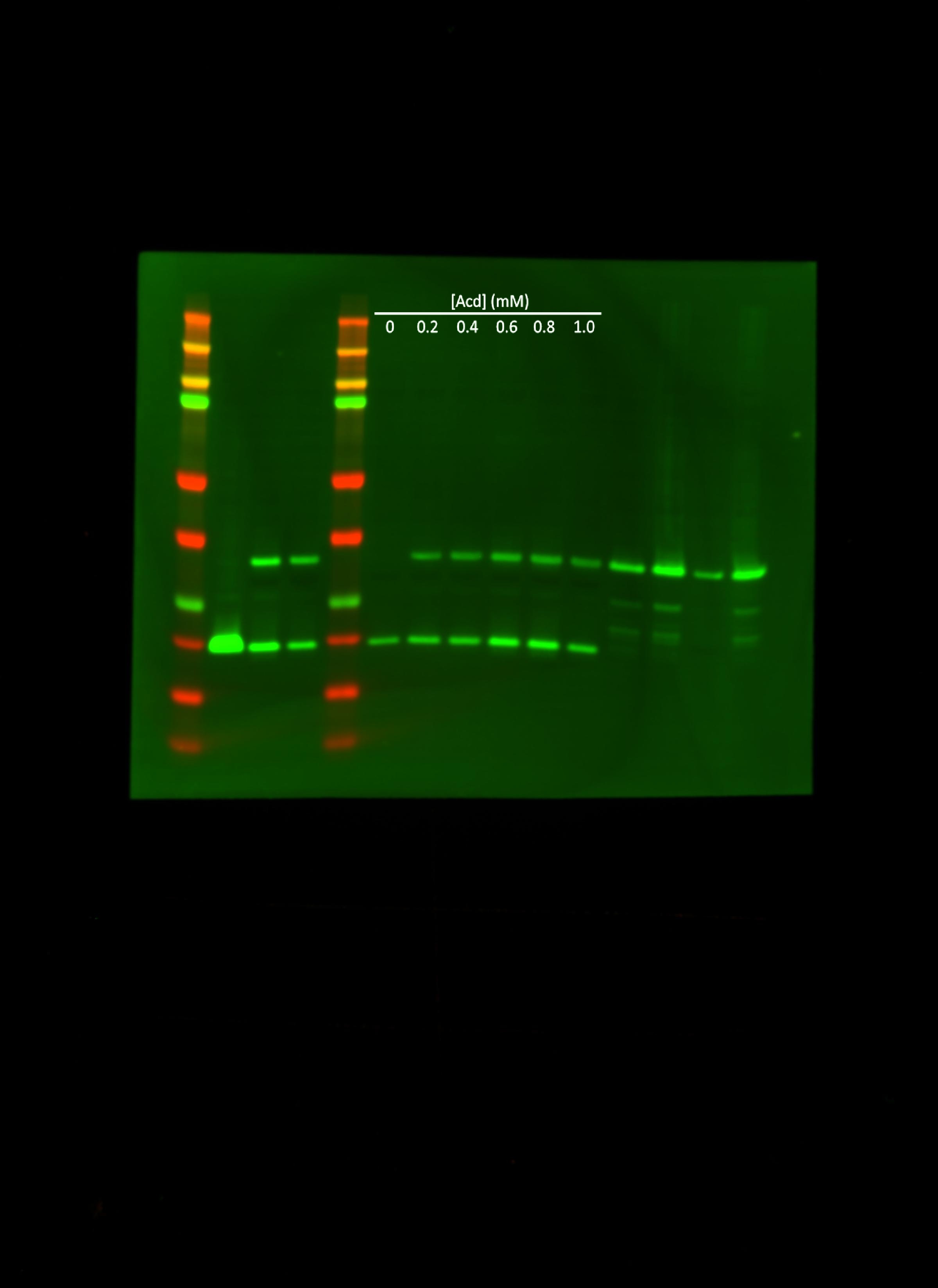

Supplement: Figure 2—figure supplement 3—source data 1. [file elife-110161-fig2-figsupp3-data1.zip › hHv1-K125TAG Acd WB Labeled.jpg]

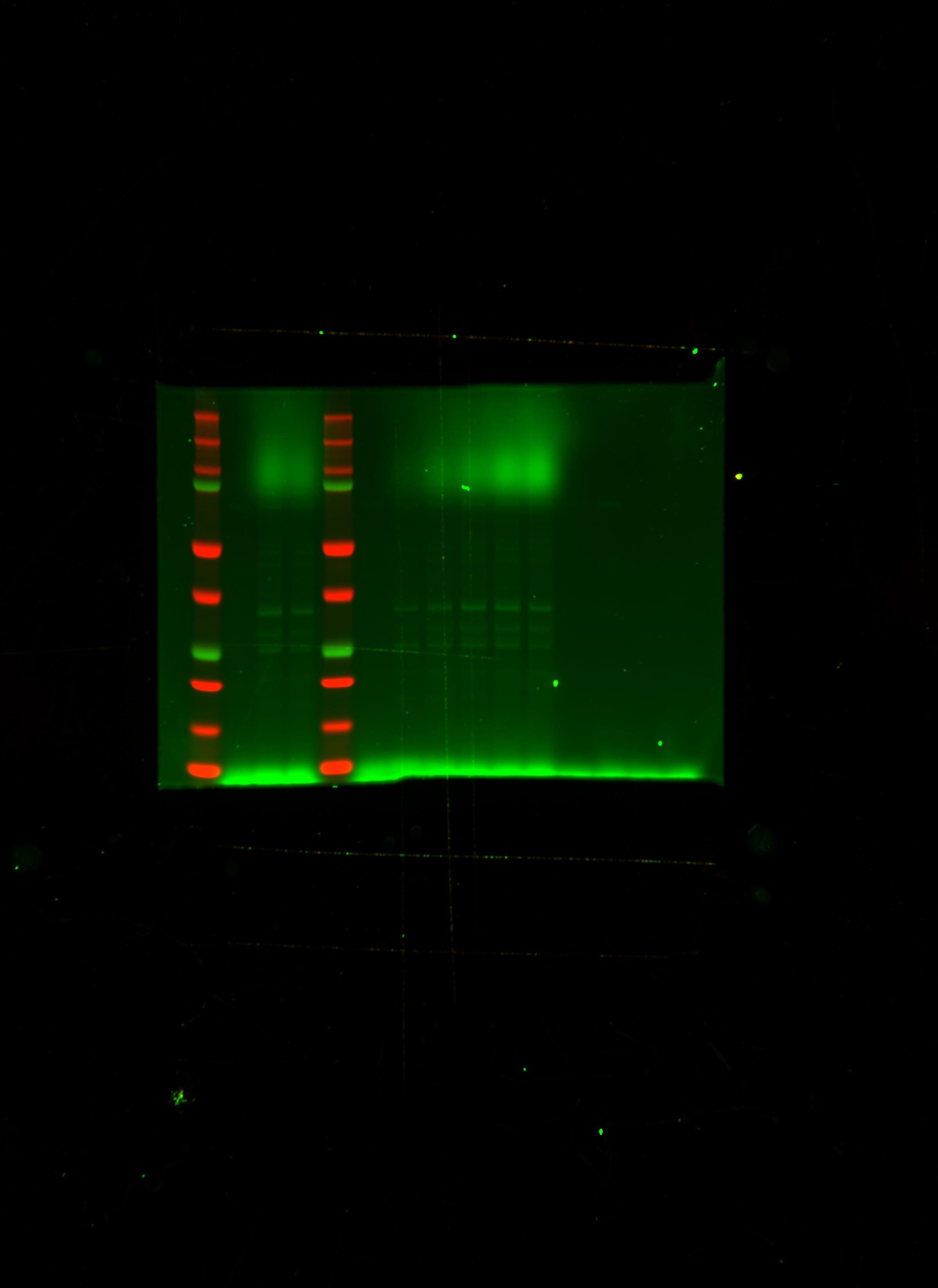

Supplement: Figure 2—figure supplement 3—source data 2. [file elife-110161-fig2-figsupp3-data2.zip › hHv1-K125TAG Acd fluorescence.jpg]

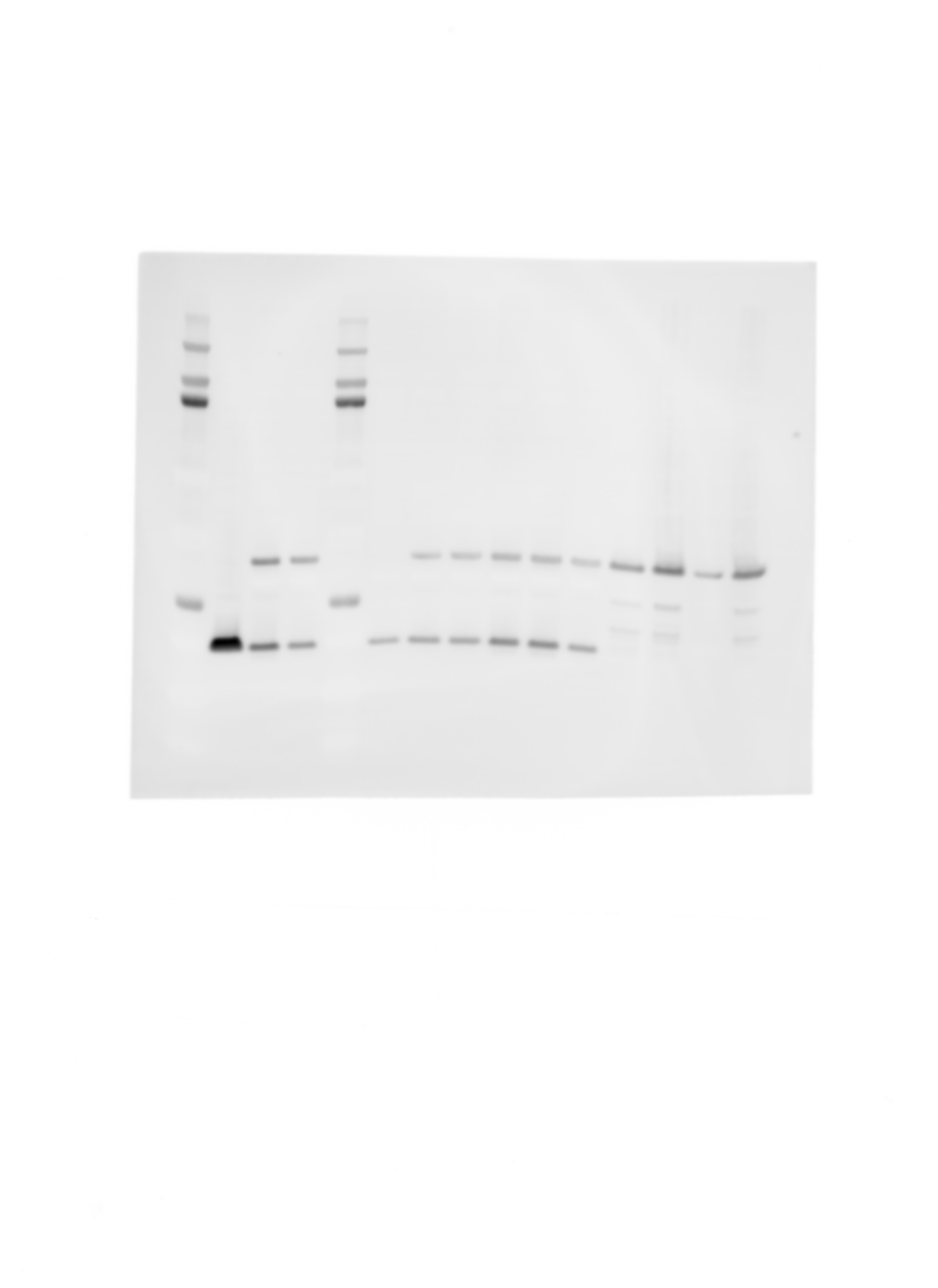

Supplement: Figure 2—figure supplement 3—source data 2. [file elife-110161-fig2-figsupp3-data2.zip › hHv1-K125TAG Acd WB.tif]

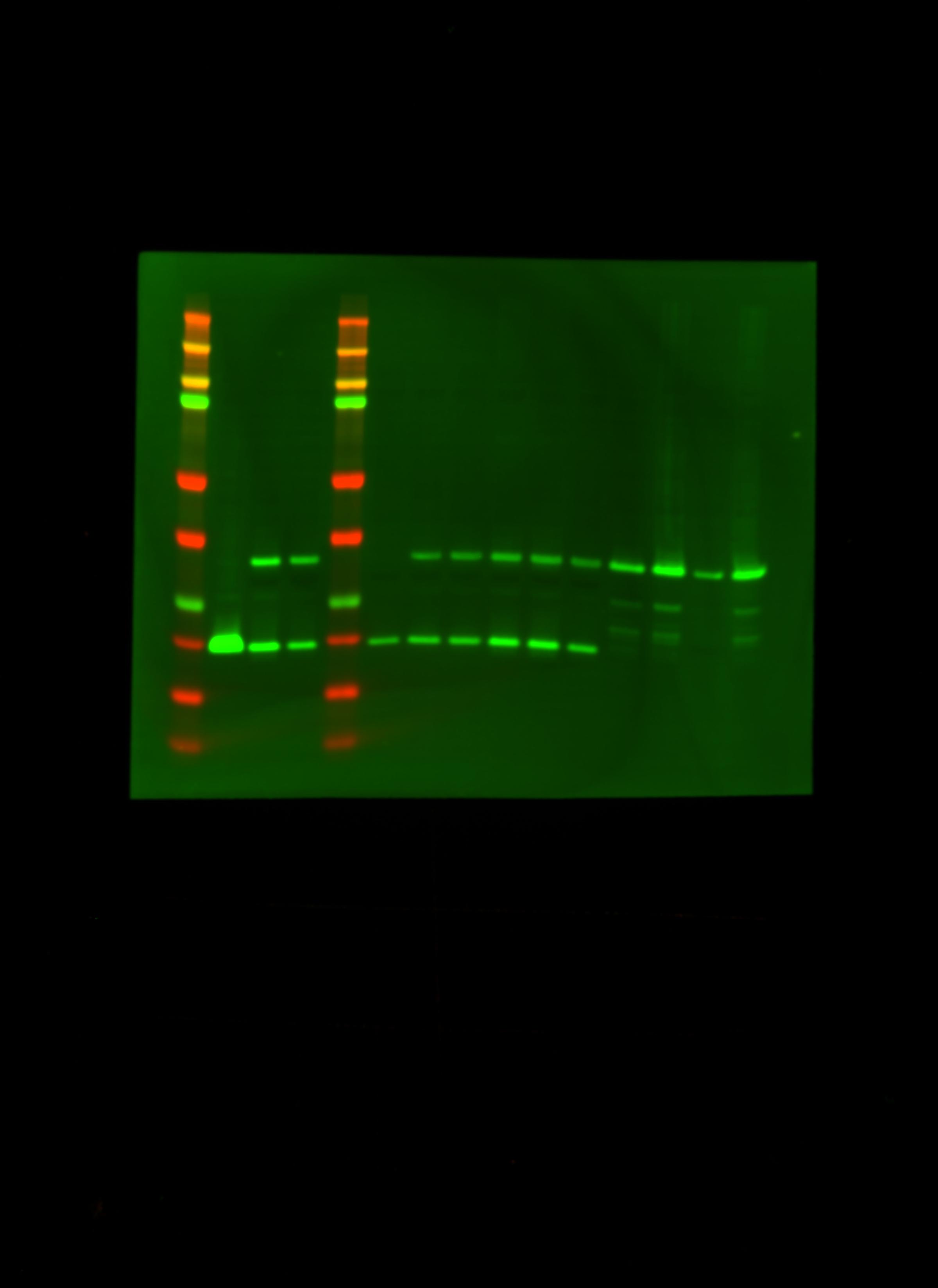

Supplement: Figure 2—figure supplement 3—source data 2. [file elife-110161-fig2-figsupp3-data2.zip › hHv1-K125TAG Acd WB.jpg]

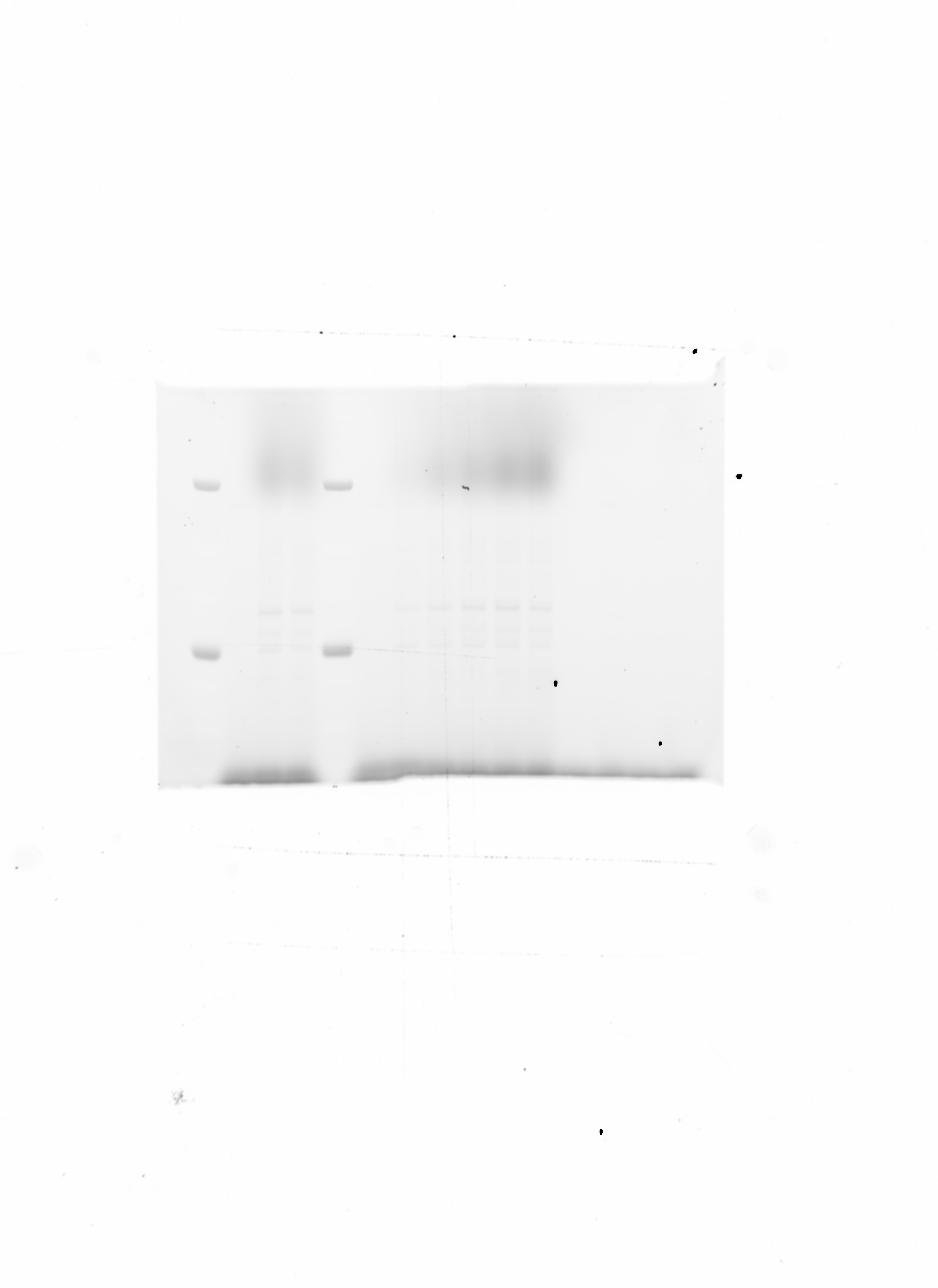

Supplement: Figure 2—figure supplement 3—source data 2. [file elife-110161-fig2-figsupp3-data2.zip › hHv1-K125TAG Acd fluorescence.tif]

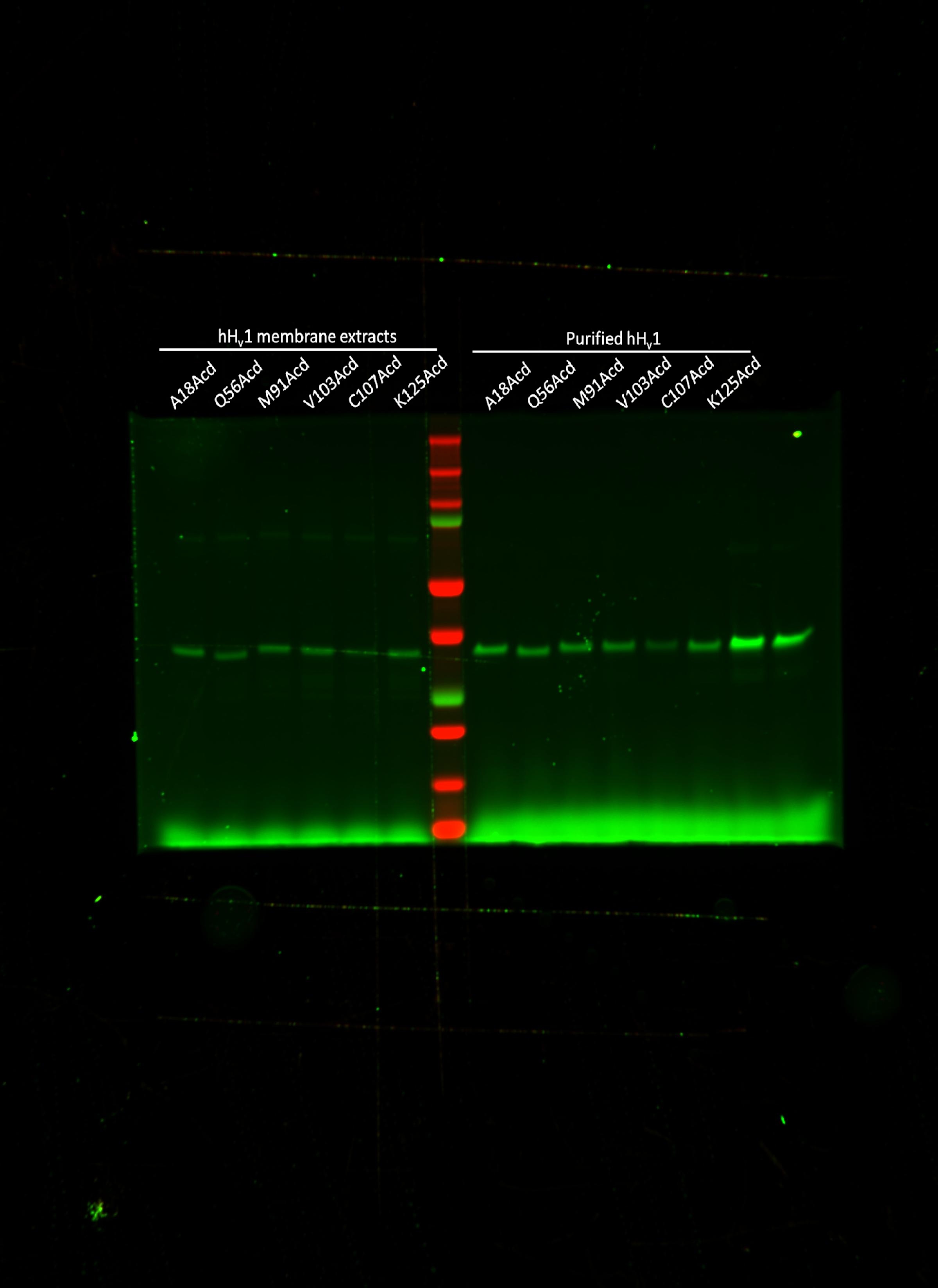

Supplement: Figure 3—source data 1. [file elife-110161-fig3-data1.zip › hHv1-Acd Purification Acd fluorescence Labeled.jpg]

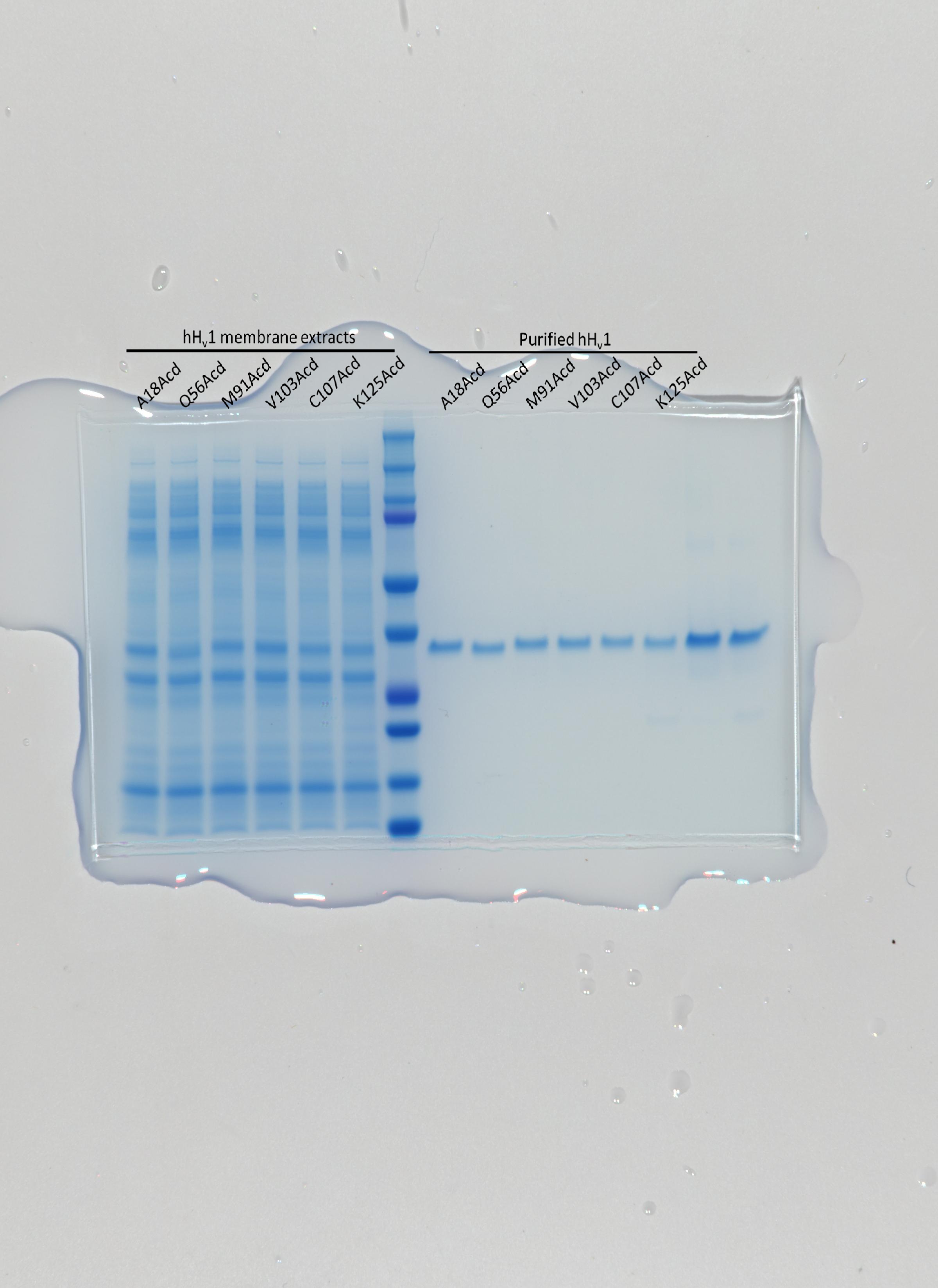

Supplement: Figure 3—source data 1. [file elife-110161-fig3-data1.zip › hHv1-Acd Purification Coomassie Labeled.jpg]

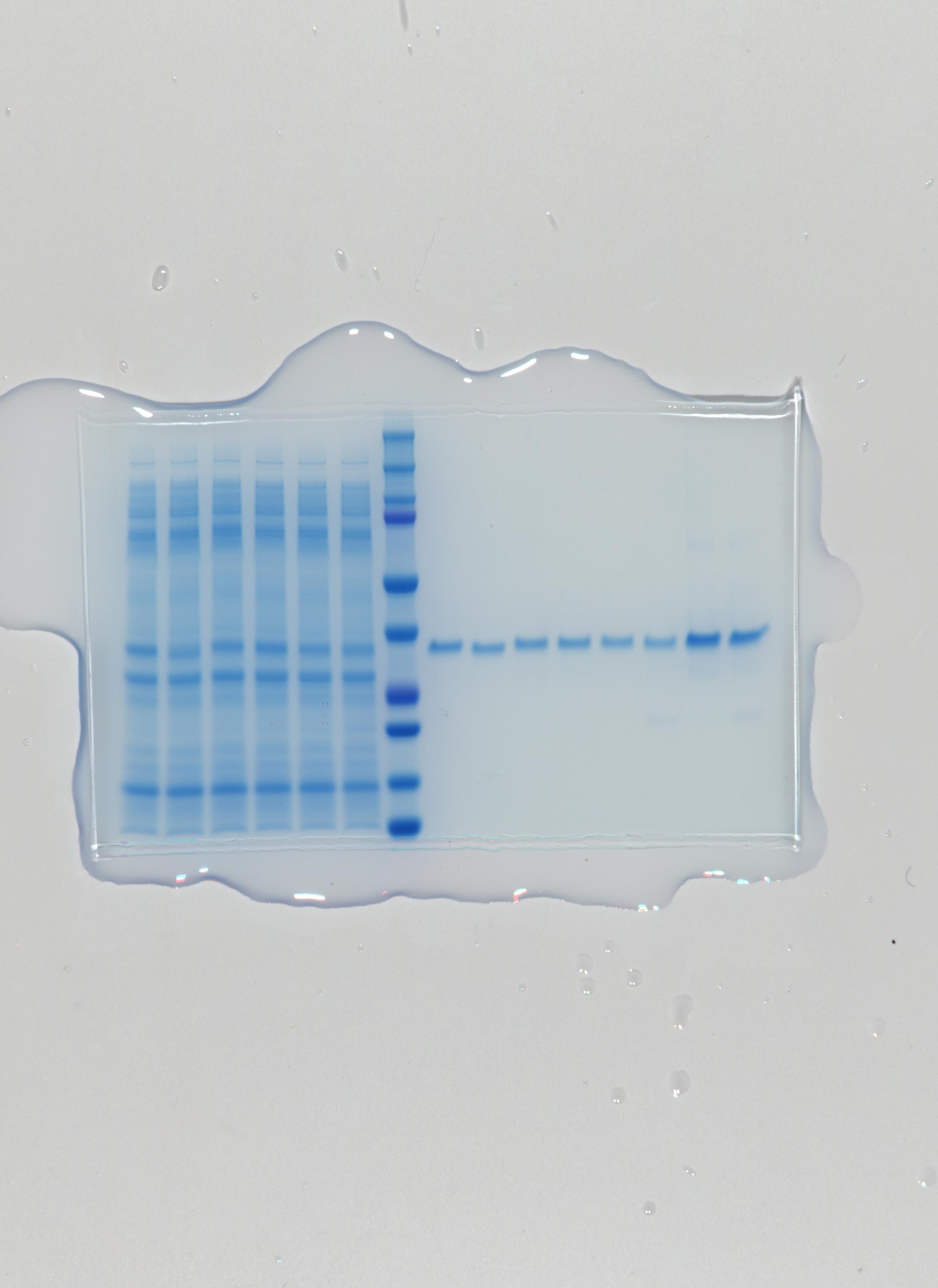

Supplement: Figure 3—source data 2. [file elife-110161-fig3-data2.zip › hHv1-Acd Purification Coomassie.jpg]

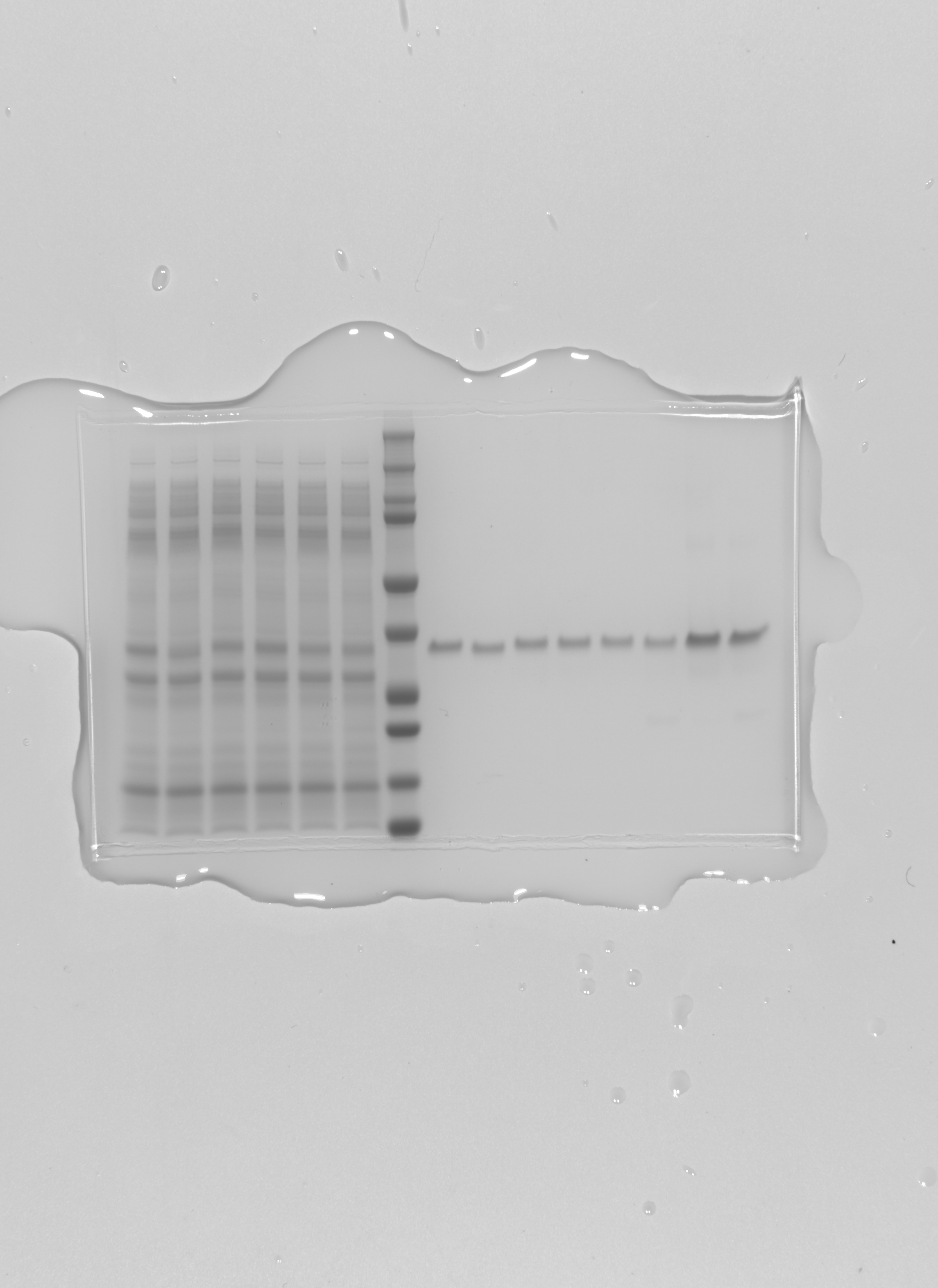

Supplement: Figure 3—source data 2. [file elife-110161-fig3-data2.zip › hHv1-Acd Purification Coomassie.tif]

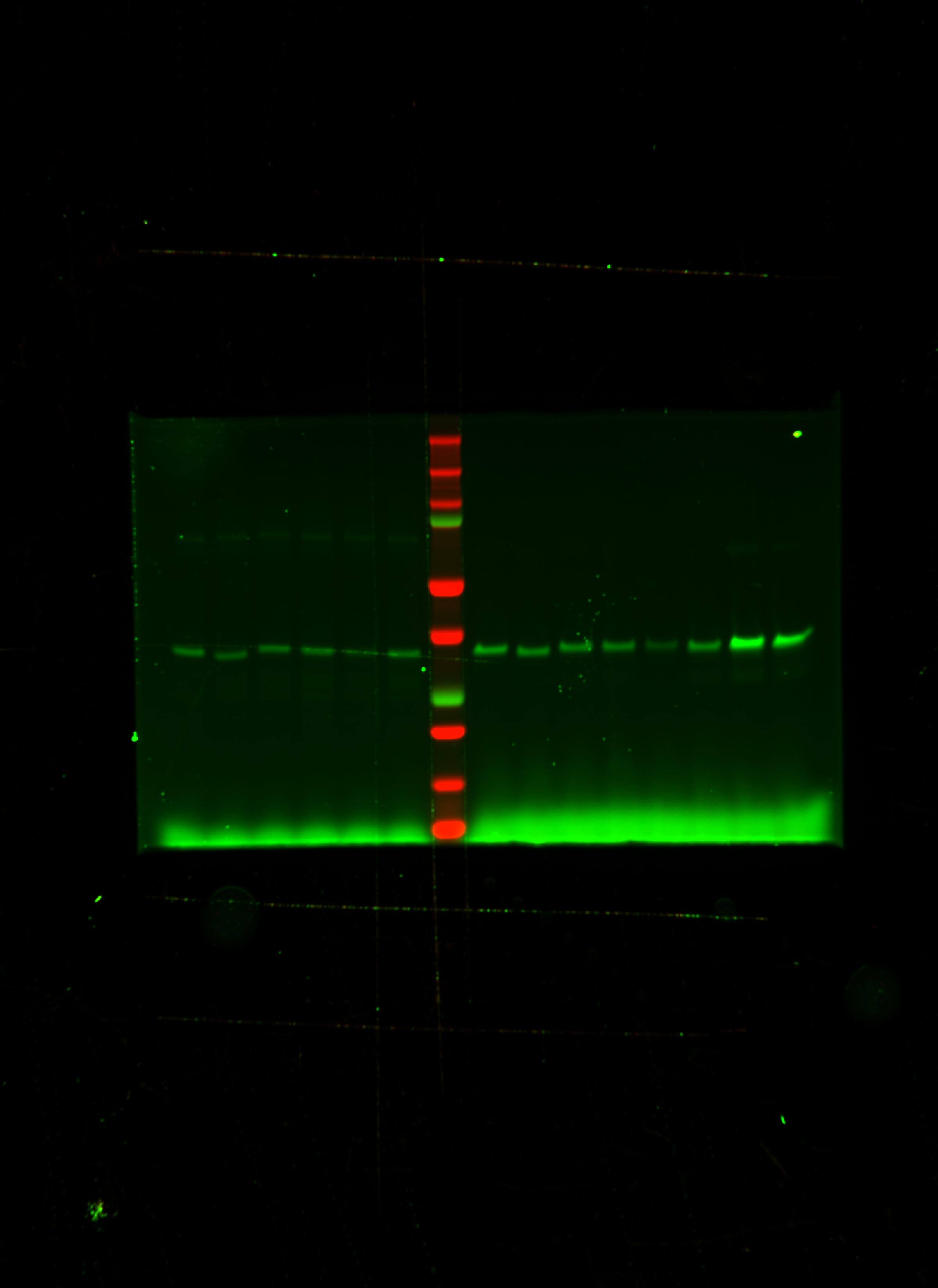

Supplement: Figure 3—source data 2. [file elife-110161-fig3-data2.zip › hHv1-Acd Purification Acd fluorescence.jpg]

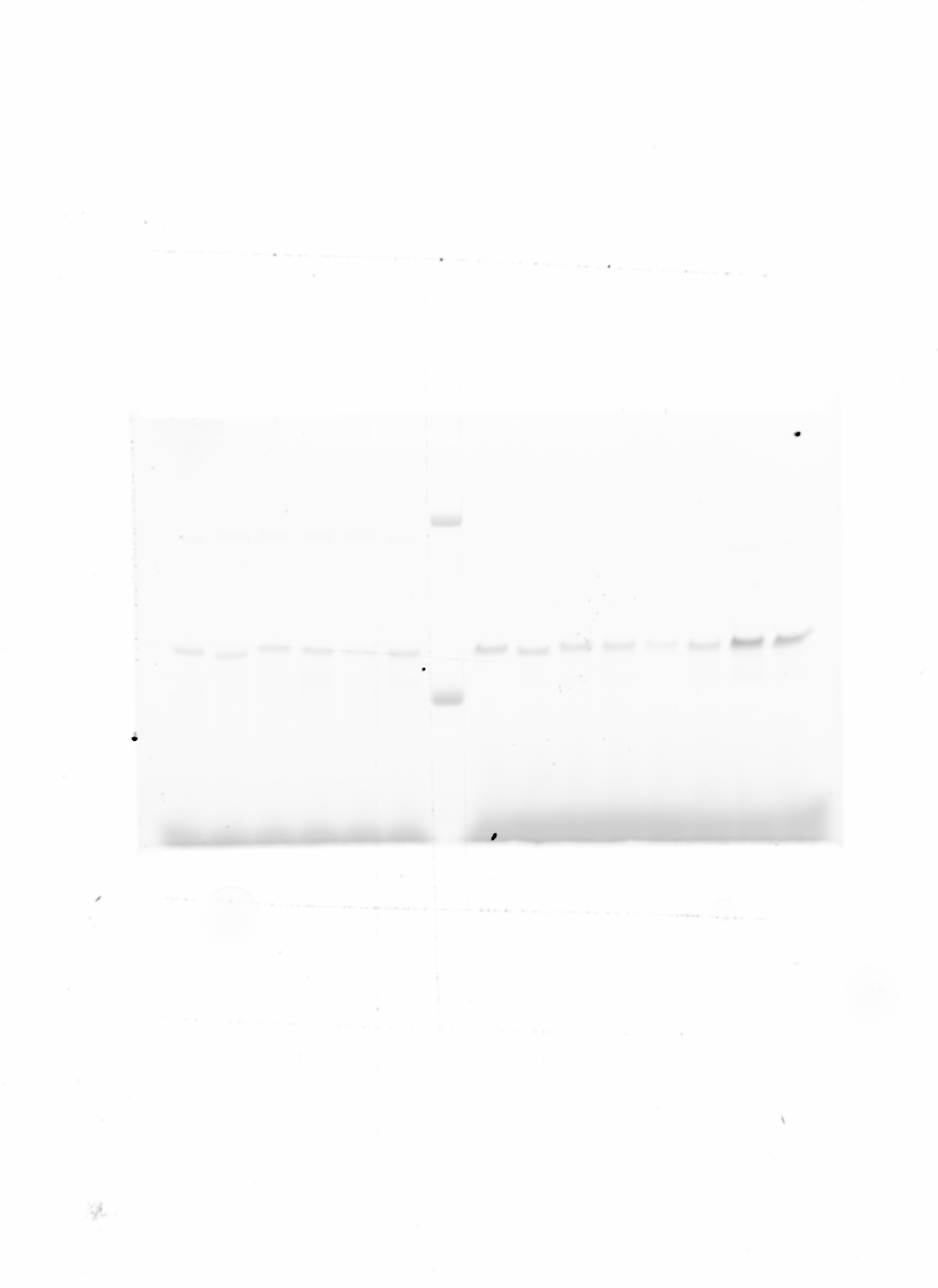

Supplement: Figure 3—source data 2. [file elife-110161-fig3-data2.zip › hHv1-Acd Purification Acd fluorescence.tif]

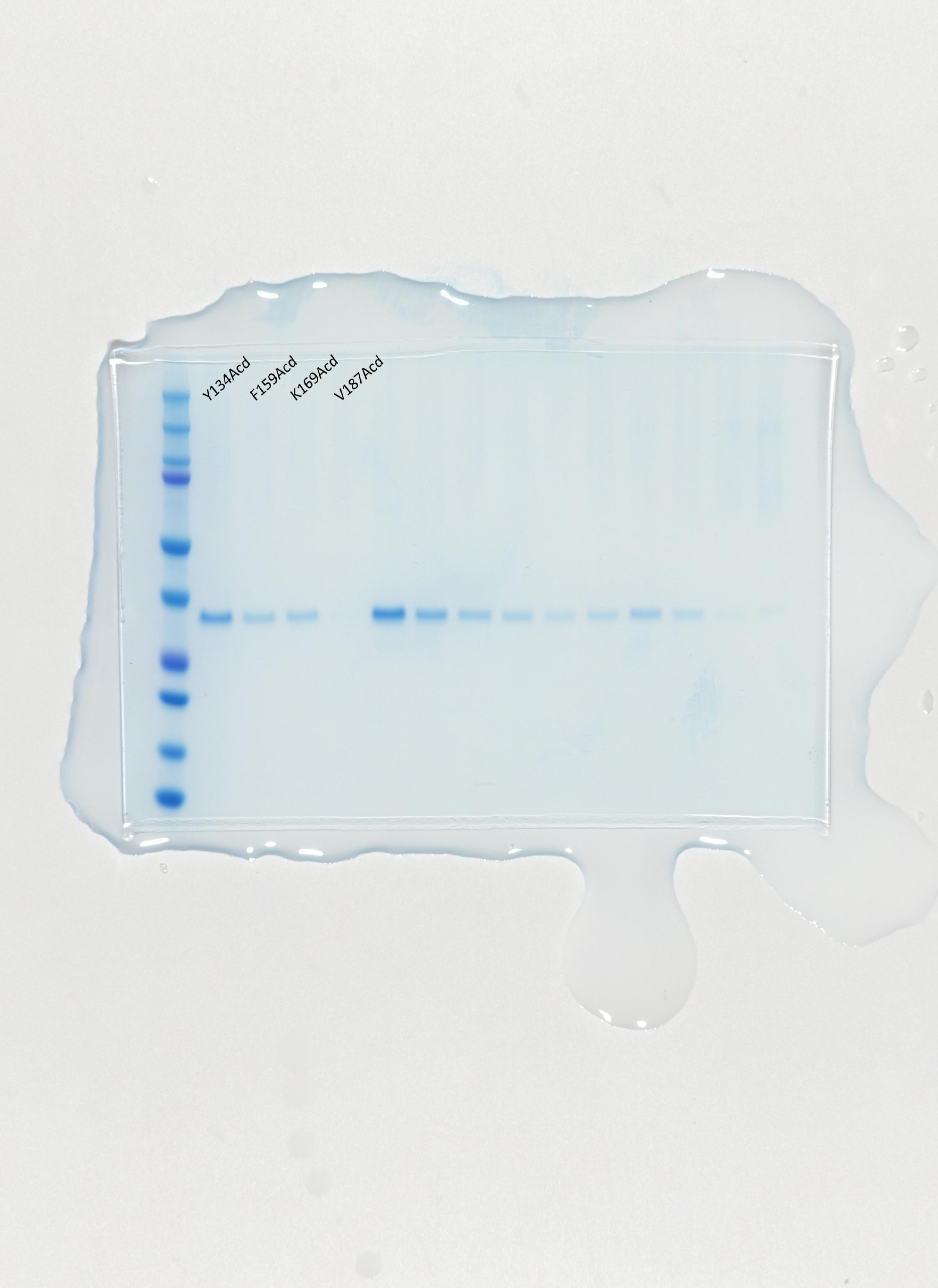

Supplement: Figure 3—figure supplement 1—source data 1. [file elife-110161-fig3-figsupp1-data1.zip › hHv1-Acd1 Coomassie Labeled.jpg]

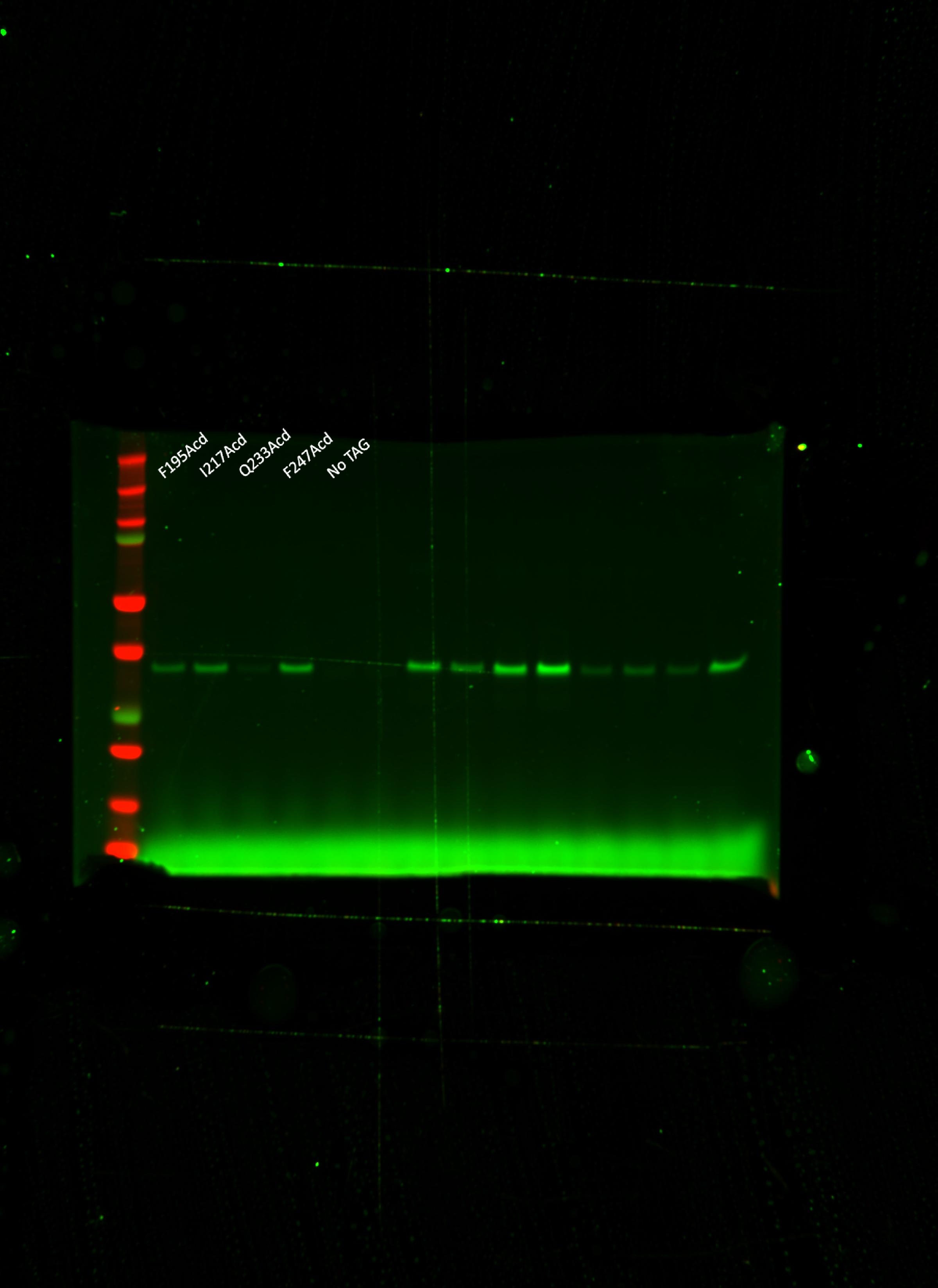

Supplement: Figure 3—figure supplement 1—source data 1. [file elife-110161-fig3-figsupp1-data1.zip › hHv1-Acd2 Acd fluorescence Labeled.jpg]

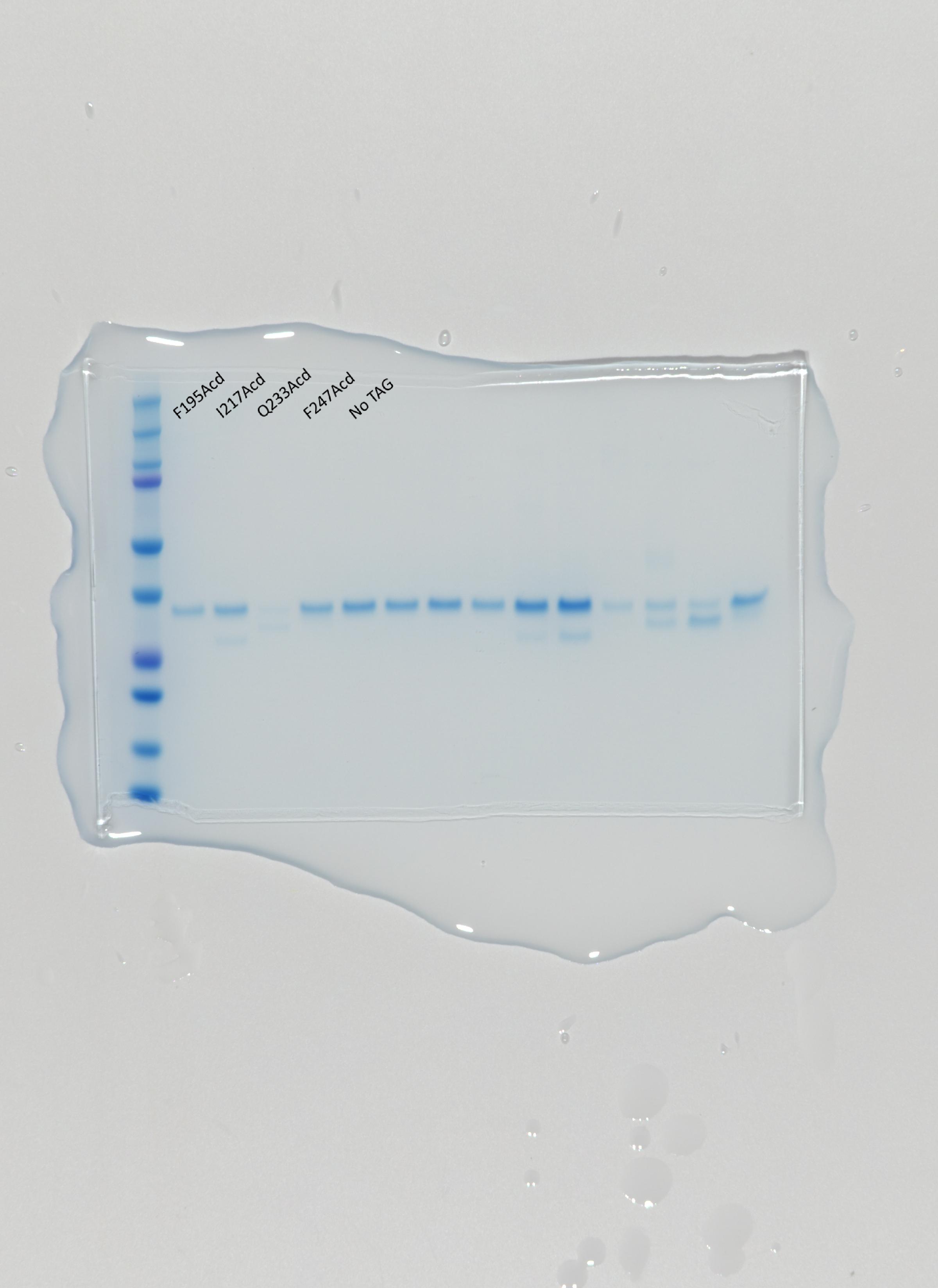

Supplement: Figure 3—figure supplement 1—source data 1. [file elife-110161-fig3-figsupp1-data1.zip › hHv1-Acd2 Coomassie Labeled.jpg]

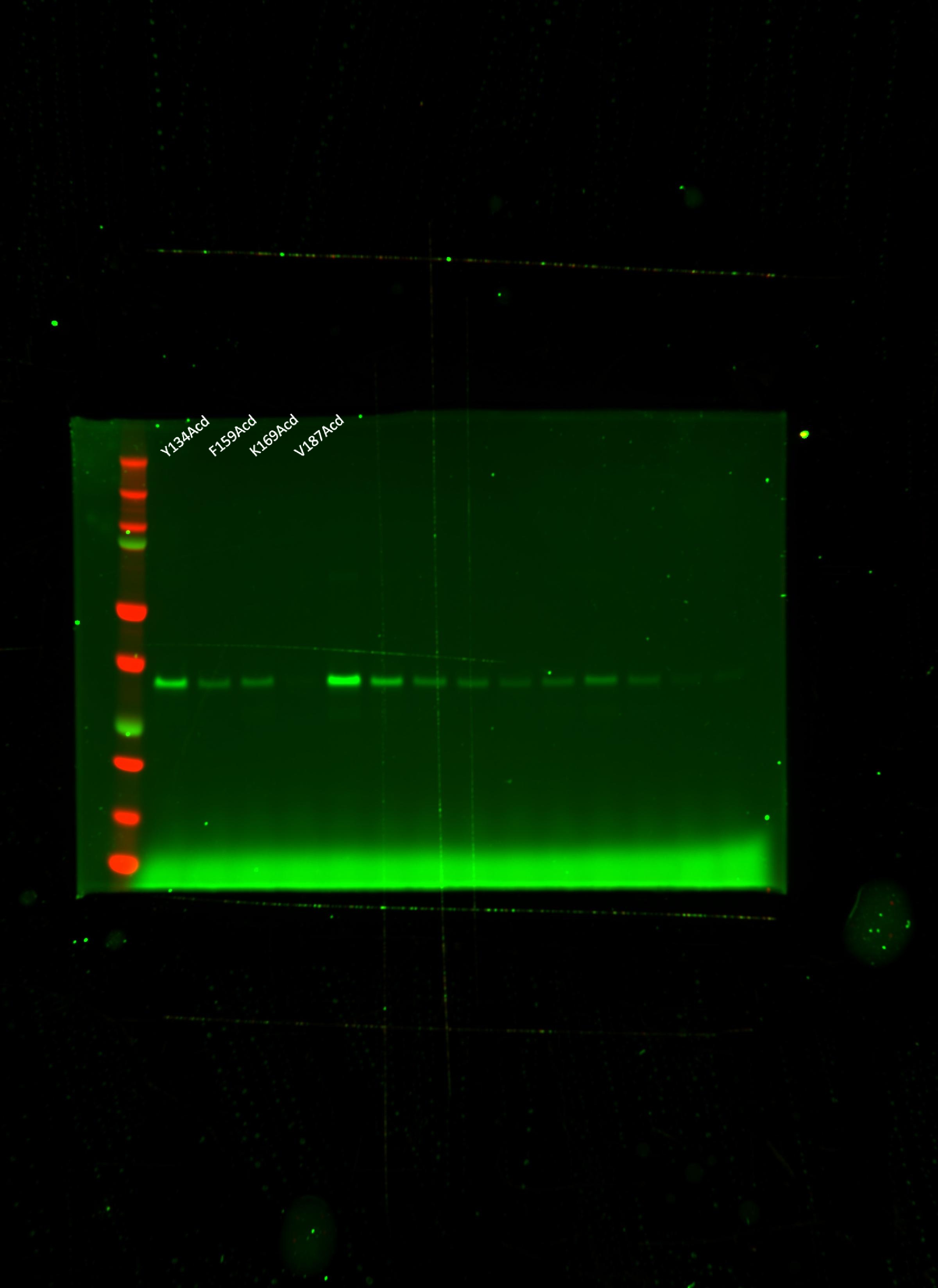

Supplement: Figure 3—figure supplement 1—source data 1. [file elife-110161-fig3-figsupp1-data1.zip › hHv1-Acd1 Acd fluorescence Labeled.jpg]

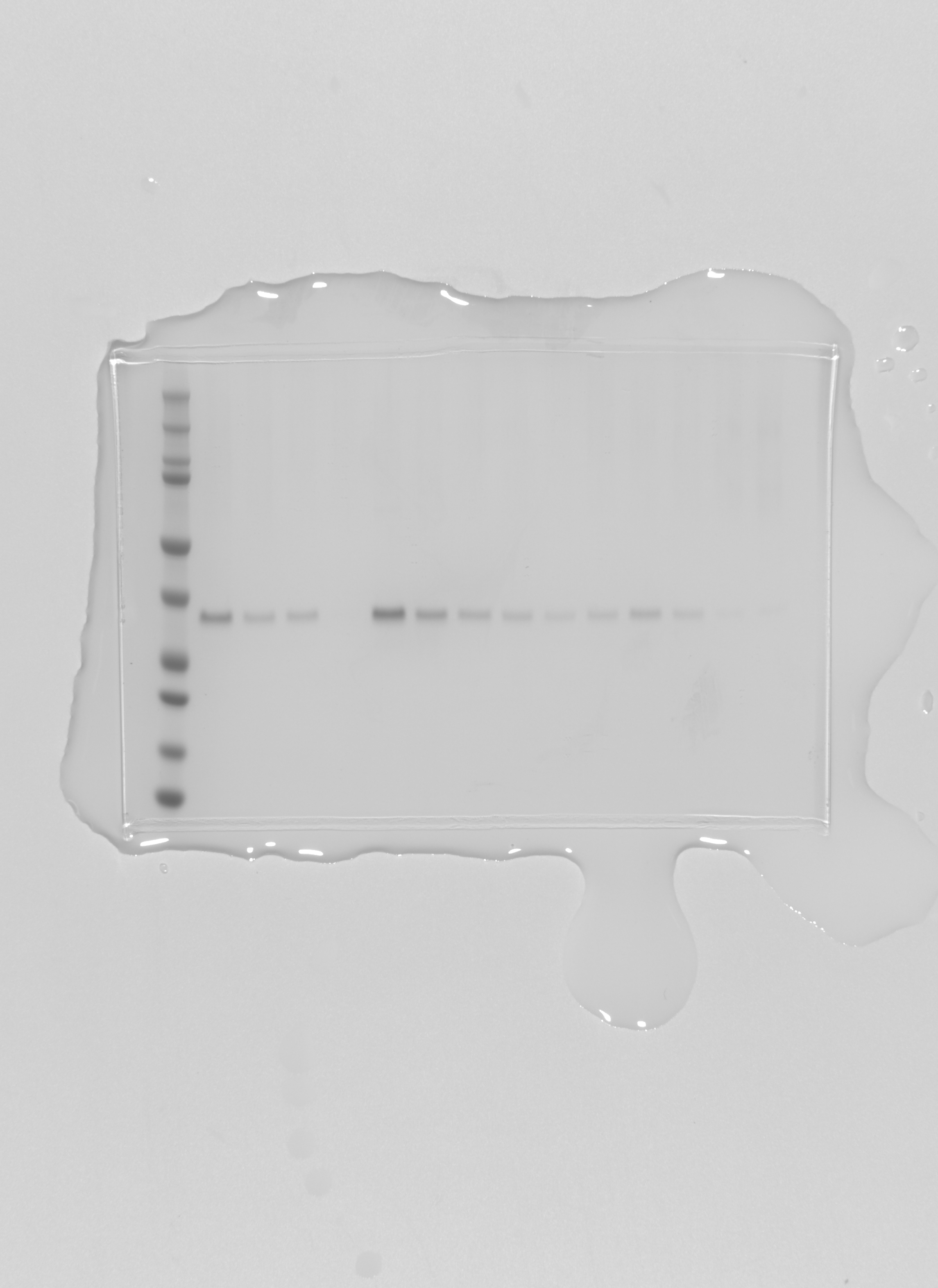

Supplement: Figure 3—figure supplement 1—source data 2. [file elife-110161-fig3-figsupp1-data2.zip › hHv1-Acd1 Coomassie.tif]

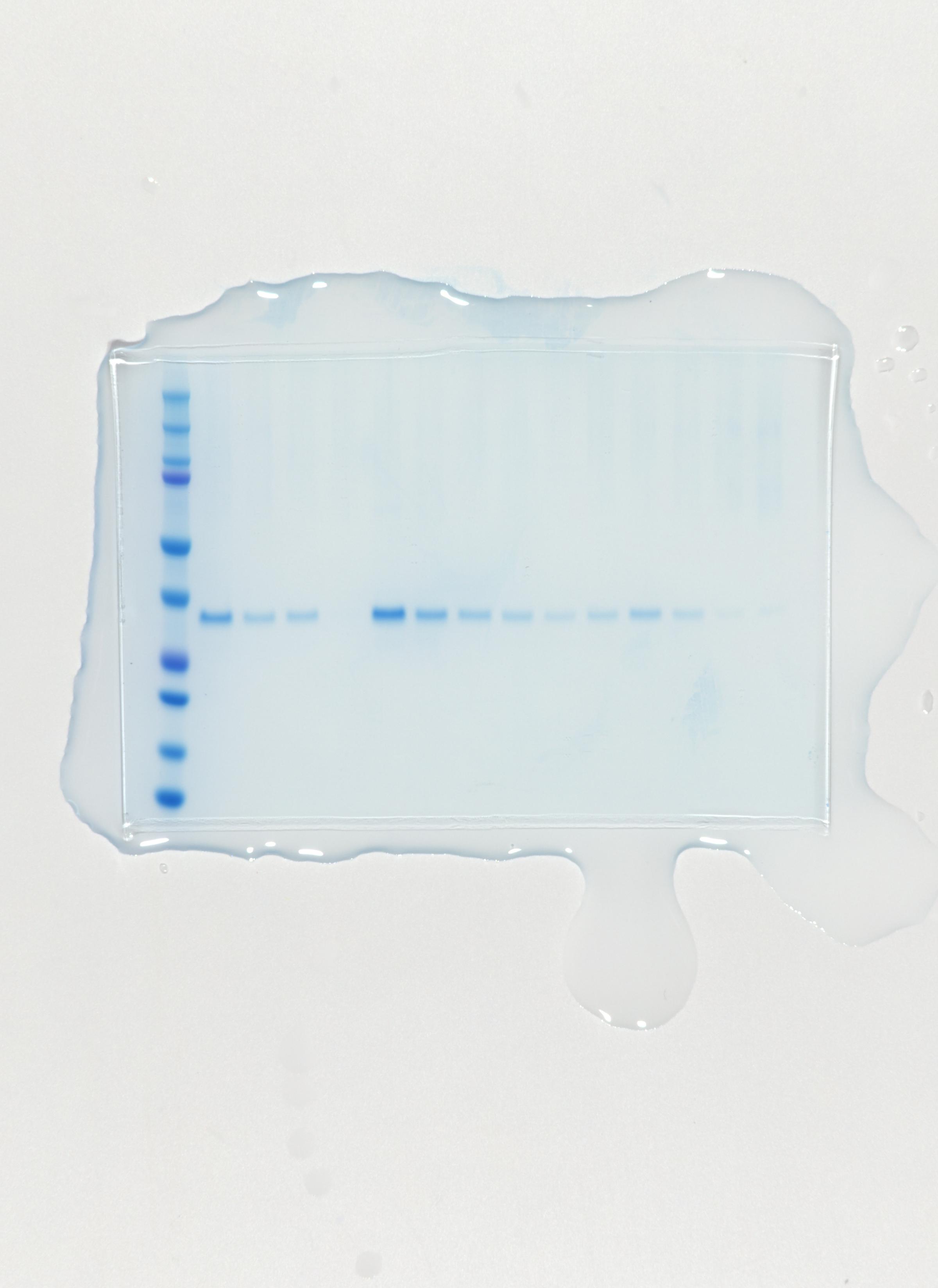

Supplement: Figure 3—figure supplement 1—source data 2. [file elife-110161-fig3-figsupp1-data2.zip › hHv1-Acd1 Coomassie.jpg]

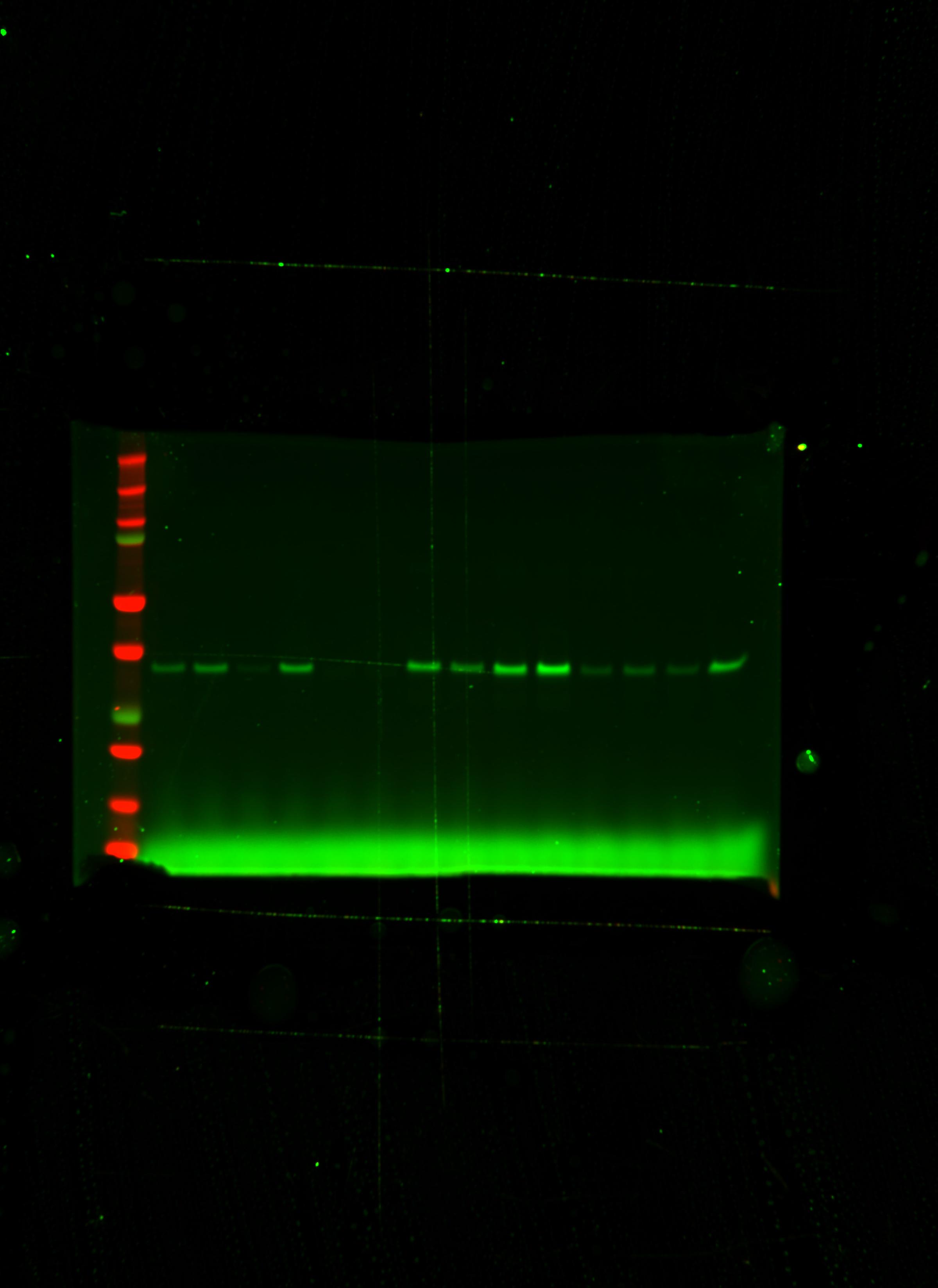

Supplement: Figure 3—figure supplement 1—source data 2. [file elife-110161-fig3-figsupp1-data2.zip › hHv1-Acd2 Acd fluorescence.jpg]

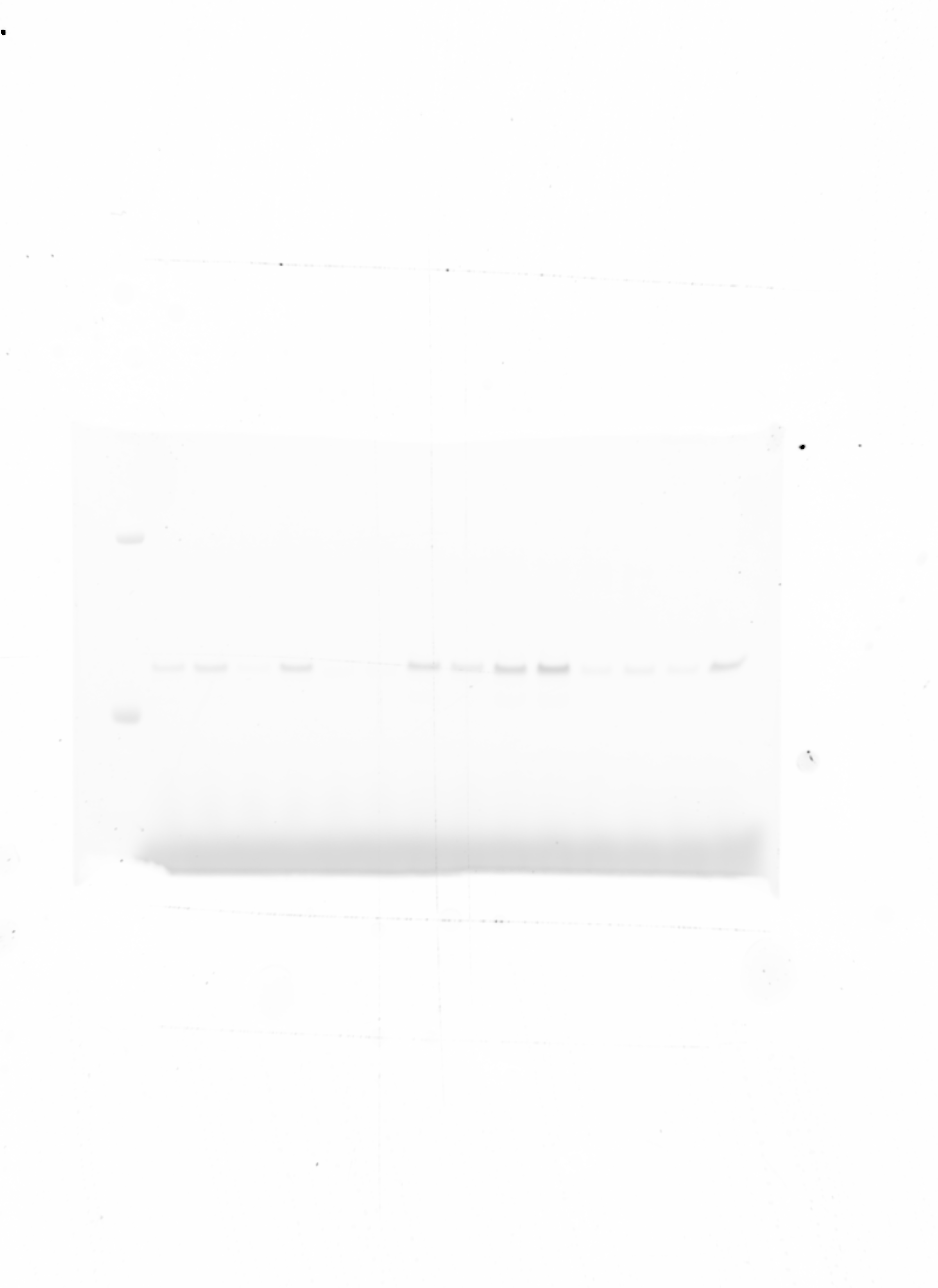

Supplement: Figure 3—figure supplement 1—source data 2. [file elife-110161-fig3-figsupp1-data2.zip › hHv1-Acd2 Acd fluorescence.tif]

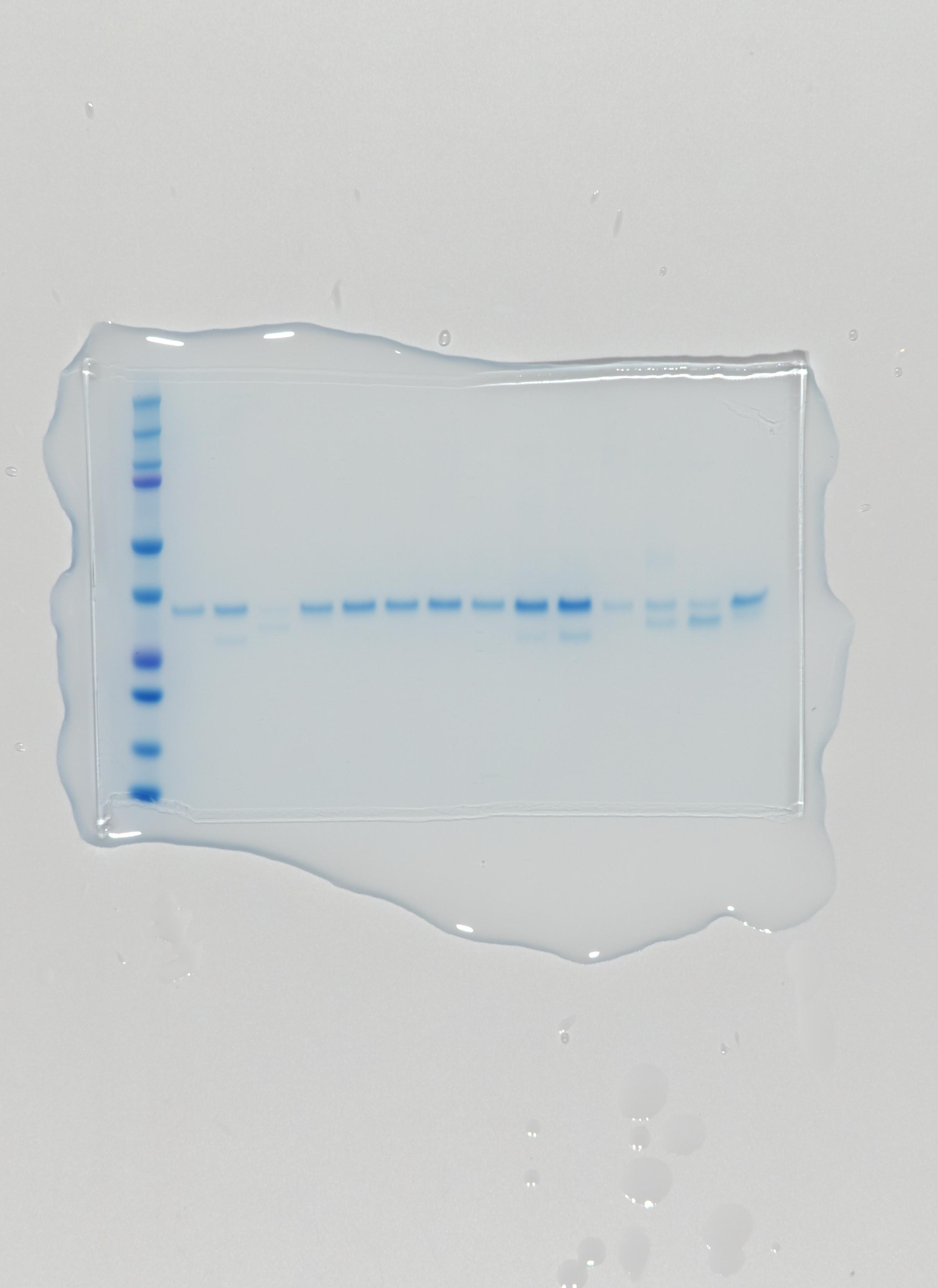

Supplement: Figure 3—figure supplement 1—source data 2. [file elife-110161-fig3-figsupp1-data2.zip › hHv1-Acd2 Coomassie.jpg]

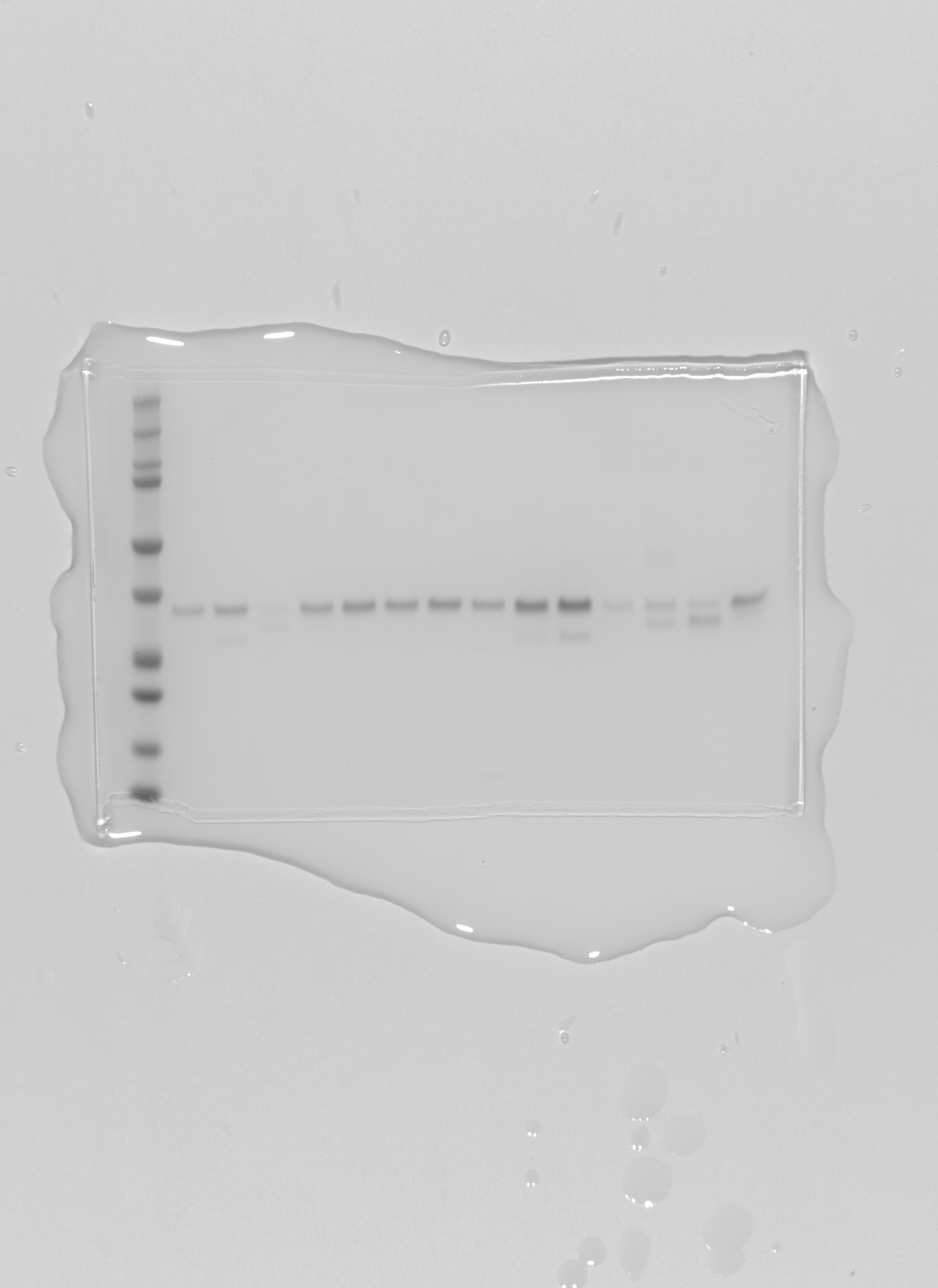

Supplement: Figure 3—figure supplement 1—source data 2. [file elife-110161-fig3-figsupp1-data2.zip › hHv1-Acd2 Coomassie.tif]

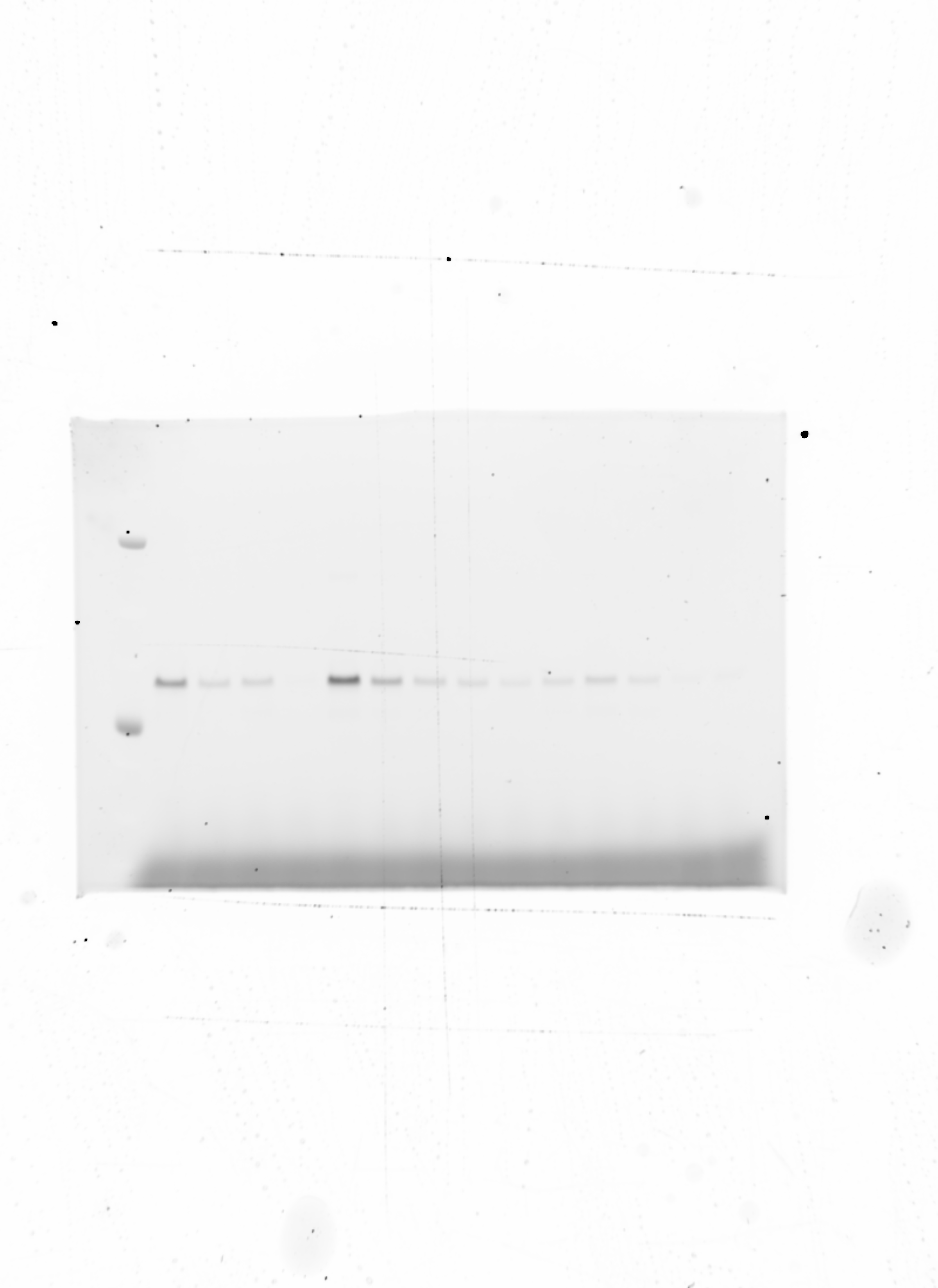

Supplement: Figure 3—figure supplement 1—source data 2. [file elife-110161-fig3-figsupp1-data2.zip › hHv1-Acd1 Acd fluorescence.tif]

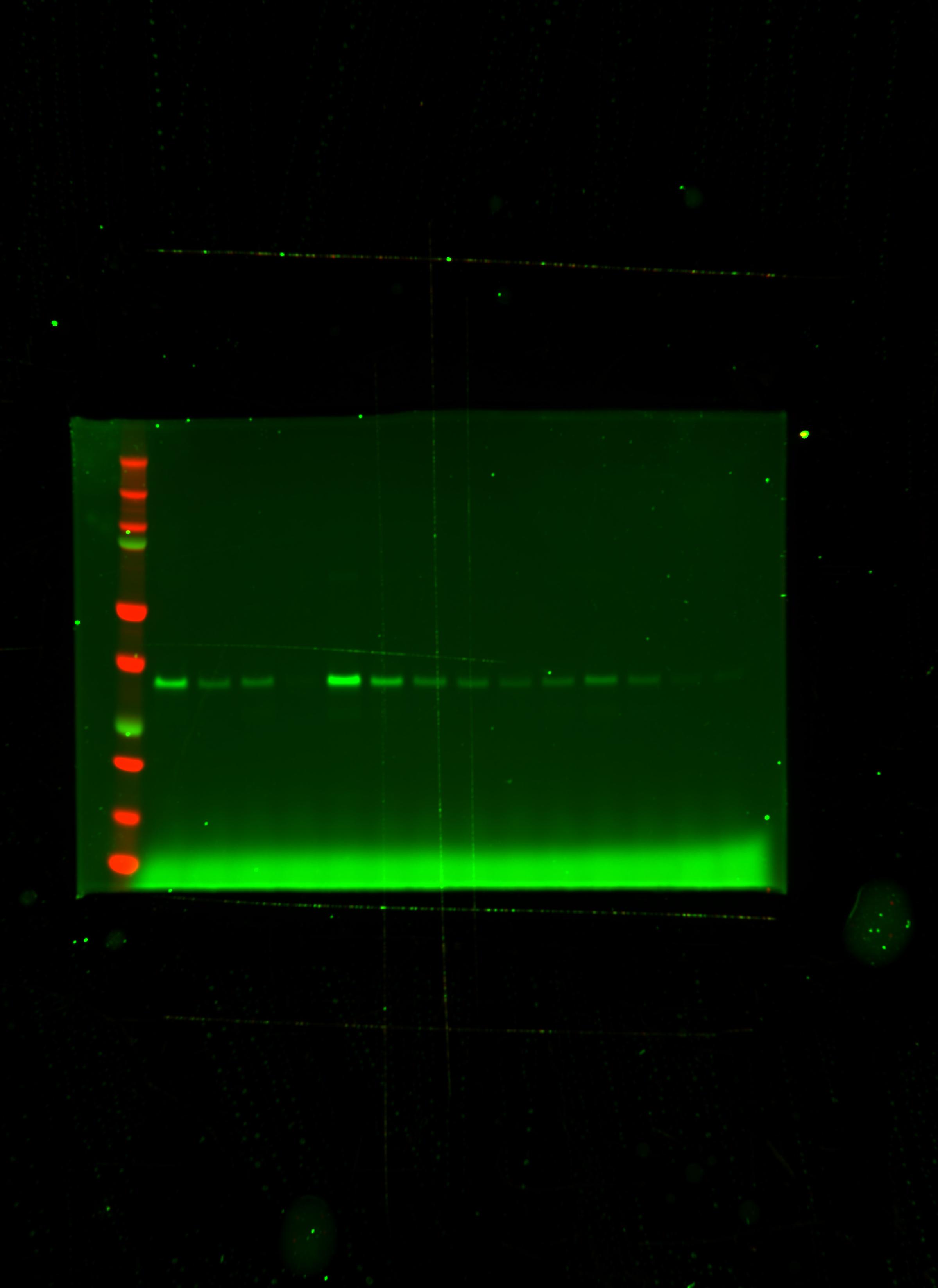

Supplement: Figure 3—figure supplement 1—source data 2. [file elife-110161-fig3-figsupp1-data2.zip › hHv1-Acd1 Acd fluorescence.jpg]
